# Structural and dynamic basis of NOD2 tandem CARD association and NOD1/2–RIP2 signaling complexes

**DOI:** 10.1371/journal.pcbi.1014311

**Published:** 2026-05-29

**Authors:** Jitendra Maharana, Aritra Bej, Debasish Biswal, Debashis Panda, Arjun Sharma

**Affiliations:** 1 Institute of Biological Chemistry, Academia Sinica, Taipei, Taiwan; 2 Taiwan International Graduate Program (TIGP), Chemical Biology and Molecular Biophysics (CBMB), Academia Sinica, Taipei, Taiwan; 3 Institute of Bioinformatics and Structural Biology, College of Life Sciences and Medicine, National Tsing Hua University, Hsinchu, Taiwan; 4 Department of Chemistry, University of California, Davis, California, United States of America; 5 Department of Pharmacology, University of California, Davis, California, United States of America; 6 Institute of Molecular Biology, Academia Sinica, Taipei, Taiwan; 7 Taiwan International Graduate Program (TIGP) - Interdisciplinary Neuroscience (INS), Academia Sinica, Taipei, Taiwan; 8 College of Life Science, National Taiwan University, Taipei, Taiwan; 9 DBT-APSCS&T, Centre of Excellence for Bioresources and Sustainable Development, Kimin, Arunachal Pradesh, India; 10 Department of Chemistry and Biochemistry, Purdue University Fort Wayne, Fort Wayne, Indiana, United States of America; KU: The University of Kansas, UNITED STATES OF AMERICA

## Abstract

NOD1 and NOD2, founding members of the NOD-like receptor (NLR) family, play a crucial role in host defense against bacterial infections. Recognition of peptidoglycan-derived ligands triggers ATP-dependent oligomerization of the NACHT domain, exposing the CARD domains that recruit the adaptor protein RIP2 via CARD–CARD interactions to activate the NF-κB signaling cascade. Although NOD1/2-RIP2 interactions and RIP2^CARD^ filament assembly are established, the precise interfaces that stabilize hetero–CARD filaments remain poorly defined. Here, we integrate *in silico* structural modeling with molecular dynamics (MD) simulations to elucidate structurally compatible arrangements of NOD1–RIP2 and NOD2–RIP2 hetero–CARD filaments. Our results reveal that NOD1^CARD^ subunits form a structurally compatible homomeric scaffold via canonical (type-I–III) interfaces, accommodating multiple tiers of RIP2^CARD^ rings at both filament termini. Meanwhile, the NOD2 tandem CARDs adopt multiple discrete conformations, reflecting a more intricate structural mechanism. In stable filament conformations, tandem CARDs converge at the type-II interface, with RIP2^CARD^ rings stacking onto CARDa (top-down) and CARDb (bottom-up) interfaces, highlighting the structural role of NOD2^CARDb^ in RIP2-mediated CARD–CARD interaction. *In silico* mutagenesis, involving charge-reversal and alanine scanning of key interfacial residues, disrupts NOD1–RIP2 and NOD2–RIP2 interactions at both top-down and bottom-up interfaces, leading to rapid interface destabilization within 0.1–0.4 μs of simulation. Together, these results reveal conserved and receptor-specific mechanisms governing NOD1/2–RIP2 CARD–CARD interactions and provide deeper structural and dynamic insights into the complex structural mechanisms for NLR-mediated inflammatory signaling.

## Author summary

NOD1 and NOD2 are cytosolic immune receptors that sense bacterial peptidoglycans and start inflammation by recruiting the adaptor protein RIP2. This recruitment relies on small signaling domains called CARDs, which nucleate and assemble into filament-like polymers that activate the NF-κB pathway. Although NOD1/2 and RIP2 hetero–CARD filaments are known to be critical for immune signaling, their structures have been challenging to resolve experimentally. As most CARD domains share a very similar structural fold, and the hetero-filaments look much like homomeric ones, cryo-EM averaging can mask subtle differences between subunits. To address this issue, we used structural modeling and molecular dynamics simulations to map the likely architectures of NOD1–RIP2 and NOD2–RIP2 hetero–CARD filaments. Our results show that NOD1 CARDs can form a stable homomeric scaffold that can accommodate RIP2^CARD^ rings at both ends of the filament. In contrast, NOD2 contains two CARDs that adopt multiple conformations and form a structurally compatible arrangement involving a type-II interface, with the second CARD (CARDb) providing the additional binding surface for RIP2^CARD^. Together, these computational results reveal both shared and receptor-specific mechanisms by which NOD1 and NOD2 drive RIP2 filament formation during immune signaling.

## 1. Introduction

Innate immunity, as an evolutionarily conserved system, serves as the primary defense against infections and relies on several sets of germline-encoded receptors known as pattern recognition receptors (PRRs). These receptors recognize pathogen-associated molecular patterns (PAMPs) or host-derived danger-associated molecular patterns (DAMPs) [1] and initiate receptor-specific signaling that drives the production and release of pro-inflammatory cytokines and chemokines. Based on their cellular location, structural fold, and pathogen specificity, PRRs are classified into five major families: Toll-like receptors (TLRs), NOD-like receptors (NLRs), RIG-I-like receptors (RLRs), C-type lectin receptors (CLRs), and AIM2-like receptors (ALRs) [2,3]. Among these, NLRs are cytoplasmic and act as intracellular surveillance molecules. There are 22 known NLRs in humans, while mice possess 34 receptors [4–8].

As founding members of the NLR family, NOD1 and NOD2 share striking structural and functional similarities [9,10]. Both receptors show tripartite domain organization – with a single N-terminal caspase recruitment domain (CARD) in NOD1 and tandem CARDs in NOD2, followed by a NACHT domain (also known as the nucleotide-binding oligomerization domain; NOD) and a C-terminal ligand-binding domain with a differing number of leucine-rich repeat (LRR) motifs [11]. NOD proteins sense bacterial peptidoglycan (PGN) fragments; NOD1 recognizes γ-D-glutamyl-meso-diaminopimelic acid (iE-DAP) [12,13], whereas NOD2 recognizes muramyl dipeptide (MDP) [14–17]. Recognition of these ligands by NOD1/2^LRRs^ induces a conformational change following ATP-dependent self-oligomerization of the NACHT [8,17–20]. This self-oligomerization may regulate the exposure of the CARD(s), thereby facilitating the transmission of downstream signals through the adaptor protein, receptor-interacting serine/threonine-protein kinase 2 (RIP2), via CARD–CARD interactions, which, in turn, activate the NF-κB signaling cascades [21–33].

Recent cryo-EM studies report that the formation of RIP2^CARD^ filaments involves both NOD1^CARD^ and NOD2 tandem CARDs contributing to their nucleation [30,31], in a configuration similar to that of other CARD filaments [34–36]. However, the structure of NOD1/2^CARDs^ in complex with RIP2^CARD^ remains unresolved. As a result, the interaction interfaces and critical residues involved in the assembly of the hetero–CARD scaffold remain unclear. Structural heterogeneity within NOD1/2–RIP2 CARD–CARD filaments has further hampered the efforts to obtain a high-resolution cryo-EM structure of these complexes [30,31]. Previous experimental findings [22–26] and our earlier theoretical investigation [29,33] identified several key interfaces and residues involved in the interactions between NOD1/2 and RIP2. However, two critical gaps remain: how NOD1/2 CARDs organize the initial scaffold, and how RIP2^CARD^ subsequently interacts and forms the hetero–CARD filament scaffold. To address this significant knowledge gap, we generated multiple conformational states of the NOD1/2–RIP2 CARD–CARD filaments and performed molecular dynamics (MD) simulations. These simulations allowed us to identify key CARD–CARD interfaces and evaluate their compatibility and persistence over time. Together, these models offer valuable structural and dynamic insights into the filament architecture, providing a comprehensive framework for understanding NOD1/2–RIP2 signaling.

## 2. Results and discussion

### 2.1. NOD1^CARD^ and NOD2^CARDa^ each distinctly associate with RIP2^CARD^

Death domain (DD)–fold proteins, such as CARD and PYD, often form filaments, and this higher-order assembly is essential for downstream signaling, including induction of pro-inflammatory cytokines and chemokines [30,31,34,37–39]. Interfacial interactions within these filaments are mediated by three conserved homotypic interfaces (type-I, II, and III), allowing each domain to interact with up to six partners. In canonical DD-fold filaments, type-I interactions involve helices α1 and α4 of one subunit (type-Ia) contacting helices α2 and α3 of an adjacent subunit (type-Ib). Type-II interfaces are formed by the α4–α5 region (type-IIa) interacting with the α5–α6 loop and α6 helix (type-IIb), whereas type-III interactions involve the α3 helix (type-IIIa) engaging the α1–α2 and α4–α5 loops (type-IIIb) [40–44]. For NOD1 and NOD2, oligomerization is necessary for the recruitment of RIP2 via CARD–CARD interactions [20], thereby initiating the formation of functional filaments and facilitating the downstream transmission of danger signals. Recent cryo-EM structures of RIP2^CARD^ filament have revealed the homotypic interaction modes among CARD domains [30,31]. Based on the RIG-I^CARDs^–MAVS^CARD^ hetero–CARD filament structure [35], Pellegrini et al. proposed that activated NOD2^CARDab^ could form short helical extensions with dimensions closely matching those of RIP2^CARD^ filament [31]. Although several studies have identified key residues in the CARD domains of NOD1/2 and RIP2 [22–26,29–31,33], the exact type of interfacial interactions remains debated [27], and the detailed arrangements that stabilize filament assembly are still only partially understood.

**Fig 1:**
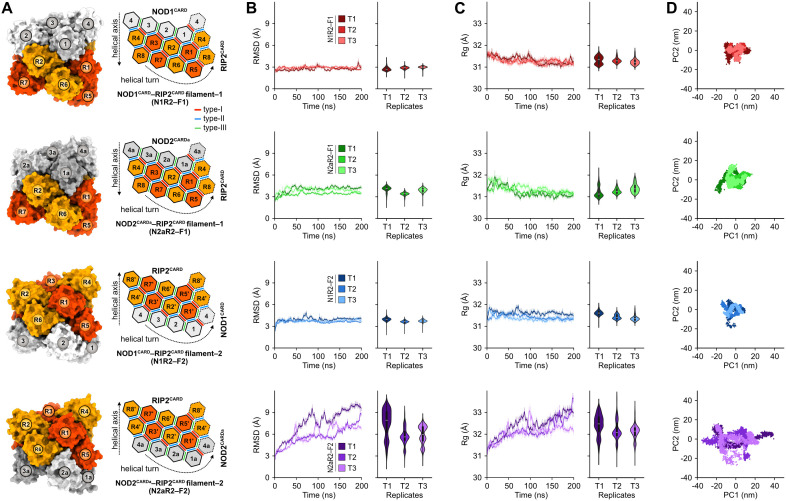
MD simulations of NOD1/2–RIP2 hetero–CARD filament models. (A) Structural models (left) and schematic representations (right) of four hetero–CARD filament assemblies: NOD1^CARD^–RIP2^CARD^ filaments (N1R2–F1 and N1R2–F2) and NOD2^CARDa^–RIP2^CARD^ (N2aR2–F1 and N2aR2–F2). Individual CARD subunits are arranged into helical layers through type-I, type-II, and type-III interfaces, as indicated in the schematics. (B) Backbone RMSD profiles over 200 ns of MD simulations for each filament model (left), with violin plots summarizing replicate-specific distributions for the three trajectories T1–T3 (right). (C) Radius of gyration (Rg) traces over 200 ns for each model (left) with corresponding replicate-wise violin plots (right), reporting overall filament compactness. (D) Principal component analysis (PC1 vs. PC2) of mainchain coordinates, illustrating the conformational space sampled by each filament model and highlighting differences in dynamic behavior among assemblies.

To investigate the interfaces and key residues that stabilize NOD1/2-RIP2 hetero–CARD filaments, we began by constructing four filament models (Fig 1A) using RIP2^CARD^ cryo-EM filament (EMD-6842) as the template, incorporating available NOD1/2–RIP2 interaction data [22–26,28–31,33]. In filament 1 (F1), one ring of NOD1^CARD^/NOD2^CARDa^ (comprising four subunits) was docked at the proximal end of the RIP2^CARD^ filament. In filament 2 (F2), the same ring was placed at the distal end (Fig 1A). Thus, N1R2/N2aR2–F1 and N1R2/N2aR2–F2 represent the top-down and bottom-up fusion models, respectively [30].

Each filament model was then simulated for up to 200 ns in triplicate to assess its structural dynamics (Table A in S1 File). Backbone root-mean-square deviation (RMSD) and radius of gyration (Rg) showed that N1R2–F1, N2aR2–F1, and N1R2–F2 models stabilized quickly, shortly after 50 ns of the production run, indicating dynamically stable filaments. In contrast, N2aR2–F2 exhibited a continuous increase in both RMSD and Rg, indicating progressive destabilization (Fig 1B and 1C). These dynamic properties were further corroborated through PCA (Fig 1D) and RMSD-based clustering (Fig AA in S2 File), which revealed significant conformational transitions in the N2aR2–F2 model. Solvent-accessible surface area (SASA) analysis further highlighted the instability of the N2aR2–F2 model, showing a SASA value (>550 nm^2^) that was higher than those of other filament models (Fig AB in S2 File).

To further assess the hetero-CARD ring-ring stability, we evaluated total number of H-bonds over time, MM/PBSA binding energies, and structural integrity across all filament complexes. H-bond analysis showed consistently stable patterns and high counts in N1R2–F1 (43.34 ± 0.99), N2aR2–F1 (35.68 ± 1.46), and N1R2–F2 (26.22 ± 5.45). However, the number of H-bonds in N2aR2–F2 gradually decreased (ranging from ~30 to ~12) over time (Fig AC in S2 File). Analysis of conformational ensembles of the filament models sampled every 50 ns further supported these observations, demonstrating that N1R2–F1, N2aR2–F1, and N1R2–F2 maintained compact, well-aligned geometries, while N2aR2–F2 adopted a distorted, extended conformation with reduced interfacial integrity (Fig AD in S2 File). MM/PBSA comparative binding scores further distinguished the stability of these interfaces based on energetic decomposition across the trajectories (T1–T3) (Table B in S1 and Fig AE in S2 Files). The top-down models N1R2–F1 and N2aR2–F1 showed favorable ΔG_bind_ (–310.63 to –338.13 kcal/mol, and –257.82 to –288.61 kcal/mol, respectively), and the bottom-up N1R2–F2 complex also remained relatively stable (ΔG_bind_ = –191.63 to –246.22 kcal/mol). By contrast, the N2aR2–F2 interface showed weak binding (ΔG_bind_ = –64.18 to –106.29 kcal/mol) due to positive Δ_Eele_ and modest ΔE_vdW_. Across the three stable models, favorable gas-phase interactions (ΔE_gas_) were partially offset by polar solvation penalties (ΔG_solvp_), particularly in N1R2–F1 and N2aR2–F1. In short, among the four filament models, only N2aR2–F2 fails to form a stable hetero–CARD assembly.

### 2.2. NOD1^CARD^, but not NOD2^CARDa^, assemble into short filament

**Fig 2:**
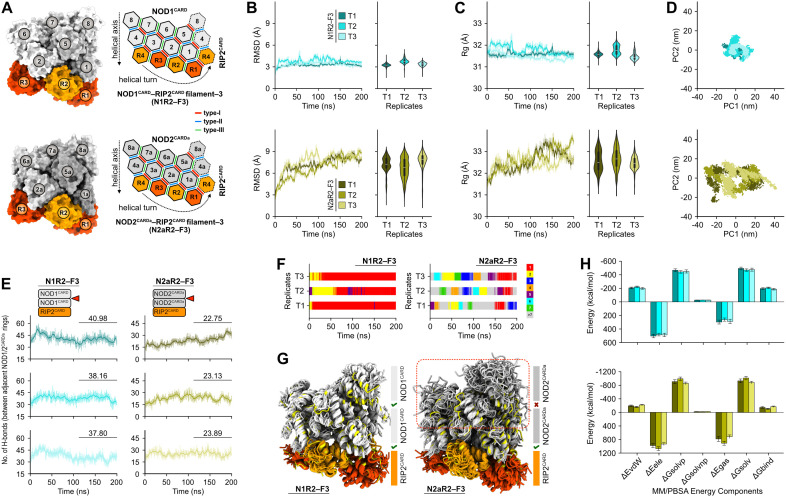
NOD1^CARD^, but not NOD2^CARDa^, forms a stable short filament. (A) Structural models (left) and schematic representations (right) of the NOD1^CARD^–RIP2^CARD^ (N1R2–F3) and NOD2^CARDa^-RIP2^CARD^ (N2aR2–F3) filament models. (B) Backbone RMSD and (C) Rg traces over 200 ns of simulation time for three independent simulations (T1–T3), with violin plots showing replicate-wise distributions. (D) Principal component analysis plots (PC1 vs. PC2) depicting conformational sampling of N1R2–F3 (top) and N2aR2–F3 (bottom). (E) Time evolution of CARD ring interfaces formed within NOD1^CARD^ and NOD2^CARDa^ homo–rings across replicates. (F) RMSD-based clustering analysis reveals distinct conformational states for each filament model; colors denote individual clusters, with red indicating the dominant state. (G) Conformational ensembles of N1R2–F3 and N2aR2–F3 derived from trajectory snapshots at 50 ns intervals; green check marks indicate compatible interfaces, whereas red crosses indicate incompatible ones. (H) MM/PBSA-derived binding free energies at homo–CARD ring interfaces.

Recent studies have suggested that NOD1 and NOD2 CARDs can oligomerize into short filaments that help recruit RIP2^CARD^ and promote subsequent filamentation [30,31]. To explore whether NOD1^CARD^ and NOD2^CARDa^ can form short filaments, we generated two additional filament models: NOD1^CARD^–RIP2^CARD^ (N1R2–F3) and NOD2^CARDa^–RIP2^CARD^ (N2aR2–F3). In these models, two tiers of NOD1^CARD^ and NOD2^CARDa^ (comprising eight subunits) were docked over a layer of RIP2^CARD^ in a top-down configuration (Fig 2A). Each model was then simulated for 200 ns in triplicate to assess the filament stability.

Backbone RMSD and Rg showed that N1R2–F3 reached equilibrium within 3–5 ns and maintained a compact structure throughout the simulation (Fig 2B and 2C). In contrast, N2aR2–F3 exhibited a rapid rise in RMSD and Rg over simulation time, indicating a progressive destabilization of the filament. PCA analysis further confirmed this difference: N1R2–F3 remained confined to a narrow conformational space with lower eigenvalues, whereas N2aR2–F3 explored a broader, more heterogeneous landscape (Fig 2D). The interfacial stability at homo–CARD ring interfaces was further evaluated by computing interfacial H-bonds over time. N1R2–F3 (NOD1-NOD1) consistently maintained a high, stable H-bond count (38.98 ± 1.74), whereas N2aR2–F3 (NOD2^CARDa^–NOD2^CARDa^) showed a lower (23.26 ± 0.58) and less consistent H-bonding pattern (Fig 2E). RMSD-based clustering reinforced these observations: N1R2–F3 trajectories were dominated by a single, stable conformational cluster, while those of N2aR2–F3 sampled multiple divergent conformations (Fig 2F). Conformational ensemble analysis further confirmed that N1R2–F3 maintained a compact ring–ring arrangement, while N2aR2–F3 progressively deviated from its initial geometry (Fig 2G). MM/PBSA calculations showed that N1R2–F3 displayed consistently favorable binding (ΔG_bind_ = −187.84 to −207.35 kcal/mol), whereas N2aR2–F3 was weaker and more variable (ΔG_bind_ = −104.50 to −169.25 kcal/mol), with a 2-fold higher electrostatic penalty (Fig 2H, and Table C in S1 File). These results support a model in which NOD1^CARD^ forms a more stable ring–ring assembly, whereas NOD2^CARDa^ alone is less stable. In this context, NOD2^CARDb^ could provide an additional interface that potentially contributes to the stabilization of the filament architecture, as suggested [33].

### 2.3. NOD1^CARD^ filament structure shows bidirectional interaction with RIP2^CARD^

**Fig 3:**
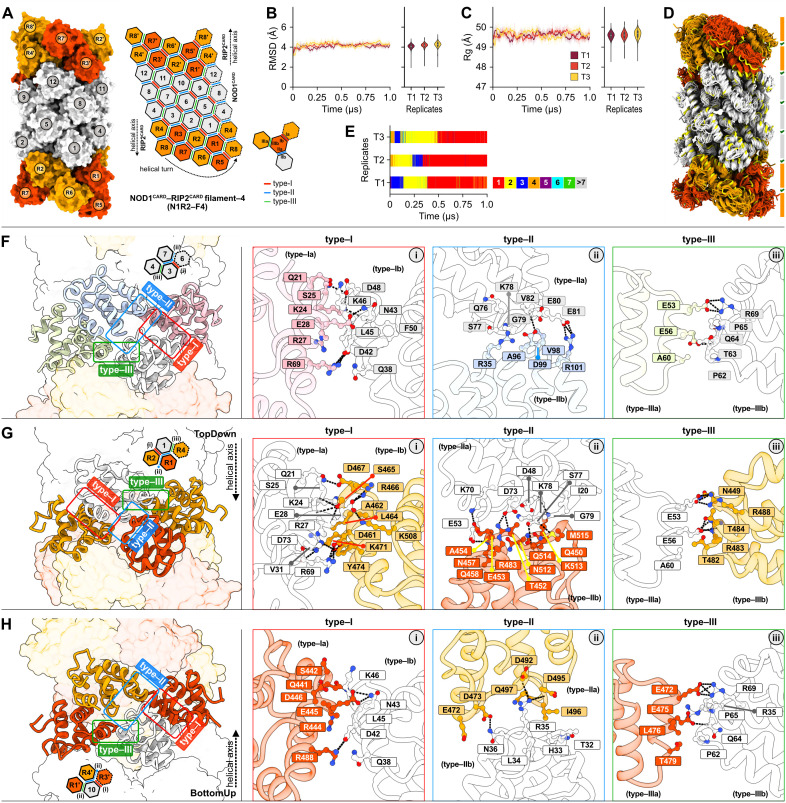
NOD1^CARD^ short filament enables bidirectional association with RIP2^CARD^. (A) Structural model (left) and schematic diagram (right) of the N1R2–F4 filament, consisting of three helical tiers of NOD1^CARD^ subunits (light gray) capped by RIP2^CARD^ at both top-down and bottom-up termini (orange/orange-red). (B) Backbone RMSD and (C) Rg plots of the N1R2–F4 over time for three independent simulations (T1-T3), with violin plots summarizing replicate-specific distributions. (D) Conformational ensemble of N1R2–F4 obtained from trajectory snapshots, illustrating overall filament stability. (E) RMSD-based clustering analysis over time, highlighting distinct conformational states; red denotes dominant clusters. (F-H) Representative CARD–CARD interfaces within the filament: (F) NOD1–NOD1, (G) NOD1–RIP2 at the top-down terminus, and (H) NOD1–RIP2 at the bottom-up terminus. Insets highlight key residue–residue contacts at (i) type-I (red), (ii) type-II (blue), and (iii) type-III (green) interfaces. NOD1^CARD^ (light gray) and RIP2^CARD^ (orange/orange red) in color cartoons, key interacting residues as ball-and-stick models with labels, and H-bonds as black dashed lines.

To characterize the integrity of the NOD1^CARD^ short filament and the association of RIP2^CARD^ rings at both ends, we constructed the N1R2–F4 model (Fig 3A) and performed cumulative 3 μs MD simulations (three independent replicates). Analysis of RMSD and Rg over time, along with snapshots from the conformational ensemble of the trajectories, showed that the N1R2–F4 maintained structural stability throughout the simulations (Fig 3B and 3D). RMSD-based clustering further indicated that a predominant, thermodynamically stable conformation emerged after ~500 ns (Fig 3E). We quantified the inter-ring H-bonds across the trajectories and calculated the corresponding MM/PBSA comparative binding scores to evaluate the stability of NOD1^CARD^ homomeric rings compared to NOD1^CARD^–RIP2^CARD^ interfaces at both the top-down and bottom-up termini (Table D in S1 and Fig B in S2 Files). All three interfaces exhibited stable interactions throughout the simulations. The NOD1–NOD1 CARD–CARD interface formed an average of 38.44 ± 1.03 H-bonds (Fig BA in S2 File), while the NOD1–RIP2 interfaces had an average of 48.40 ± 2.97 H-bonds at the top-down interface and 30.99 ± 3.33 at the bottom-up interface (Fig BB and BC in S2 File). MM/PBSA analysis of the N1R2–F4 model showed that all ring–ring interfaces displayed favorable binding free energies (ΔG_bind_) (Table D in S1 and Fig BD–BF in S2 Files). For the NOD1–NOD1 ring interface (Fig BD in S2 File), all replicates showed consistently negative ΔG_bind_ (−242.27 to −260.53 kcal/mol), indicating robust self-association driven by tightly packed complementary contacts. The NOD1–RIP2 top-down heteromeric CARD interface (Fig BE in S2 File) exhibited the most favorable ΔG_bind_ (−331.44 to −359.75 kcal/mol), driven by strong ΔE_vdW_ and cohesive ΔE_ele_, aligning with higher H-bond counts (Fig BB in S2 File). In contrast, the bottom-up NOD1–RIP2 interface (Table D in S1 and Fig BF in S2 Files) demonstrated a moderately favorable ΔG_bind_ (−203.64 to −278.81 kcal/mol), with reduced yet favorable ΔE_vdW,_ and ΔG_solvp_ contributions, consistent with fewer stabilizing H-bonds (Fig BC in S2 File). Collectively, these results indicate that all ring–ring interfaces are structurally compatible but differ in relative stability, with the top-down NOD1–RIP2 interface being the most stable.

### 2.4. Homodimeric multi-interface interactions stabilize the NOD1^CARD^ short filament

Previous studies have demonstrated the self-dimerization of NOD1^CARD^ [29,45], and our simulations show that NOD1^CARD^ self-assembles through canonical (type-I–III) interfaces. H-bond analysis across the trajectories and interfaces revealed persistent and reproducible interactions. On average, type-I interface formed 5.74 ± 0.94 H-bonds, whereas type-II and type-III interfaces formed 3.53 ± 0.34 and 3.38 ± 0.54 H-bonds, respectively (Fig CA–CC in S2 File), indicating stable interactions at each interface. Consistent with these H-bonding patterns, MM/PBSA calculations showed that each interface type exhibits a distinct energetic signature (Table E in S1 and Fig CD–CF in S2 Files). Type-I was the most stable interface (ΔG_bind_ = −23.42 to −37.94 kcal/mol), type-II displayed a strong ΔE_vdW_ stabilization, resulting in a stable ΔG_bind_ (−26.80 to −38.44 kcal/mol). On the other hand, type-III, despite being comparatively weaker, remained stable (ΔGbind = −16.99 to −18.25 kcal/mol) due to moderate contributions from ΔE_vdW_ and ΔG_solv_, which were sufficient to offset the unfavorable ΔE_ele_ (Table E in S1 and Fig CF in S2 Files).

To analyze the key residues contributing to each interface, we extracted 250 snapshots from the final 500 ns of the N1R2–F4 T2 (selected based on clustering analysis; Fig 3E), and performed dynamic interaction mapping and per-residue MM/PBSA energy decomposition. The interaction analysis revealed that H-bonds, supported by several vdW contacts, predominantly stabilize these interfaces (Fig 3F). At the type-I interface, we identified six H-bonds (Q21-D48, K24-D48/F50, E28-N43, and R69-Q38/D42), two electrostatic interactions (E28-K46 and R27-D42), and multiple vdW contacts between the type-Ia and type-Ib surfaces (Fig 3Fi, Fig CG in S2 File). Type-II and type-III interfaces displayed fewer H-bonds, with three at type-II (R35-Q76, D99-E80, R101-E81) and two at type-III (E53-R69 and E56-Q64), each accompanied by several vdW contacts and a single hydrophobic interaction (Fig 3Fii and 3Fiii, Fig CH and CI in S2 File). Per-residue MM/PBSA decomposition highlighted that the type-I interface was dominated by residues K24, R27, R69, and K70 (type-Ia) and D42, N43, L45, K46, D48, and F50 (type-Ib) (Fig CJ in S2 File). For type-II, the key residues were Q76, S77, K78, and G79 (type-IIa) and R35, N36, Q38, V98, and R101 (type-IIb) (Fig CK in S2 File). Lastly, type-III was characterized by residues E53 (type-IIIa) and R35, T63, Q64, P65, K67, and R69 (type-IIIb) (Fig CL in S2 File). Together, these results demonstrate that NOD1^CARD^ homodimer adopts three energetically distinct yet consistently stable CARD–CARD interfaces, each contributing a unique structural and energetic configuration within the modeled filament architecture.

### 2.5. RIP2^CARD^ binds NOD1^CARD^ filament at both ends

To characterize how RIP2^CARD^ interacts with NOD1^CARD^ filament at each end, we examined the interfacial H-bonds and performed MM/PBSA energetics assessments across all three interfaces. At the top-down terminus, all interfaces demonstrated stability with consistent patterns and higher H-bond counts: type-I formed 8.14 ± 0.35 H-bonds, type-II 5.90 ± 0.65, and type-III 5.39 ± 0.05 (Fig DA–DC in S2 File). MM/PBSA calculations further confirmed this strong interfacial stability (Table E in S1 and Fig DD–DF in S2 Files). Type-I exhibited consistently favorable ΔG_bind_ (−44.95 to −46.26 kcal/mol), largely driven by ΔE_vdW_. Type-II exhibited the strongest interaction (ΔG_bind_ = −40.54 to −54.42 kcal/mol), with contributions from both favorable ΔE_vdW_ and ΔE_ele_. Type-III interaction was weaker but remained favorable (ΔG_bind_ = −19.79 to −20.31 kcal/mol). Meanwhile, the bottom-up terminus interfaces showed comparatively weaker and more dynamic interactions. H-bond counts were consistently lower but remained reproducible across replicates: type-I formed 3.95 ± 1.34 H-bonds, type-II 3.24 ± 1.34, and type-III 3.78 ± 0.50 (Fig EA–EC in S2 File). MM/PBSA results mirrored the trends observed in H-bonding, indicating reduced stability and greater energetic fluctuations (Fig ED–EF in S2 File). All three interfaces showed favorable ΔG_bind_ values; however, type-II was most favorable (−26.46 to −40.88 kcal/mol) (Table E in S1 File). Overall, these data suggest that RIP2^CARD^ forms a structurally compatible interaction network at the top-down terminus, with type-II as the dominant contributor, whereas all three interfaces at the bottom-up terminus remain energetically viable but relatively weaker overall.

### 2.6. Bidirectional, multi-interface interactions drive NOD1–RIP2 hetero–CARD filament

The interaction between the CARDs NOD1 and RIP2 has been extensively studied over the past two decades. Manon et al. were the first to identify NOD1^CARD^ residues E53, D54, E56, and R69, and RIP2^CARD^ residues R444, R483, and R488, as critical for this interaction [22]. Later, Fridh and Rittinger showed that additional RIP2^CARD^ residues (D461, E472, E475, and D492) are pivotal for the NOD1–RIP2 interaction [24]. Boyle et al. further highlighted the key contributions of NOD1 residues E53 and D54 [25]. Mayle et al. proposed a multi-interface interaction model that includes type-I and type-III interfaces, implicating the roles of NOD1^CARD^ residues E53 and E56 (type-IIIa) and RIP2^CARD^ residues R443, R444, Y474, R483, and R488 (type-Ia) [26]. Our previous study indicated that type-II interface residues also play a critical role in hetero–CARD association [29], in line with findings by Gong and co-authors [30]. Despite these advancements, residue-level contributions across all three interfaces remain only partially understood. Because NOD1 and RIP2 interact through multiple interfaces as the filament nucleates and elongates, defining the residues that mediate each interface is essential to understanding how the hetero–CARD filament is stabilized. To this end, we performed detailed interaction mapping coupled with per-residue MM/PBSA decomposition at each interface.

Interaction analyses showed that all six NOD1^CARD^ interfaces interact with RIP2^CARD^ through distinct H-bonds, electrostatic interactions, and vdW contacts (Fig 3G and 3H; DG–DI and EG–EI in S2 File). At the top-down terminus, NOD1^CARD^ engages with RIP2^CARD^ through its type-Ia, IIb, and IIIa interfaces, which pair with the complementary type-Ib, IIa, and IIIb interfaces of RIP2^CARD^ (Fig 3G, and DG–DI in S2 File). The type-I interface is stabilized by eight H-bonds (Q21-D467, K24-D467, R27-S465/Y474, E28-S465/R466/K508, R69-D461), two electrostatic contacts (R27-D461 and D73-K471), and multiple vdW contacts (Fig 3Gi, and Fig DG in S2 File). The type-II interface exhibits five H-bonds (E53-Q458, D73-R483, S77-Q450/M515, K78-G516), two electrostatic interactions (D48-K513 and K70-E453), one hydrophobic contact (I20-M515), and additional vdW contacts (Fig 3Gii, and Fig DH in S2 File). The type-III interface features three H-bonds (E53-R488, E56-R483/T484) and two vdW contacts (Fig 3Giii, and Fig DI in S2 File). Per-residue MM/PBSA decomposition identified key energetic hotspots at each interface (Fig DJ–DL in S2 File). For the type-I, significant stabilizing energies stem from NOD1^CARD^ residues K24, R27, E28, R69, and K70 (type-Ia), as well as RIP2^CARD^ residues D461, R466, D467, K471, Y474, and K508 (type-Ib) (Fig DJ in S2 File). In the type-II interface, the stabilizing energies are primarily contributed by NOD1^CARD^ residues D48, D73, and S77 (type-IIb), and RIP2^CARD^ N457, Q458, R483, and K513 (type-IIa) (Fig DK in S2 File). For the type-III interface, the core energetic hotspots are formed by NOD1^CARD^ residues E53 and E56 (type-IIIa) and RIP2^CARD^ residues T482, R483, T484, and R488 (type-IIIb) (Fig DL in S2 File) at the top-down terminus.

In contrast, at the bottom-up terminus (Fig 3H, and Fig EG–EI in S2 File), the interfaces are inverted in orientation. Specifically, the NOD1^CARD^ type-Ib, IIa, and IIIb interfaces interact with the complementary RIP2^CARD^ type-Ia, IIb, and IIIa interfaces, respectively. At the type-I interface, stability comes through two H-bonds (D42-R488 and N37-E445), several electrostatic interactions (D42-R444, L46-E445/M446), and numerous vdW contacts (Fig 3Hi, Fig EG in S2 File). The type-II interface exhibits a more fragmented interaction pattern, characterized by transient H-bonds (R35-D473/D492/D495, N36-E472) supported by multiple vdW contacts (Fig 3Hii, and Fig EH in S2 File). The type-III interface consists of two H-bonds (Q64-E475 and R69-E472), one electrostatic interaction (R35-E475), a hydrophobic contact (P62-L476), and additional vdW contacts (Fig 3Hiii, and Fig EI in S2 File). Per-residue MM/PBSA decomposition identified the key energetic contributors to each bottom-up interface (Fig EJ–EL in S2 File). For the type-I interface, the significant contributors from NOD1^CARD^ are D42, N43, and K46 (type-Ib), while key residues from the RIP2^CARD^ include Q441, S442, K443, R444, and R488 (type-Ia) (Fig EJ in S2 File). In the type-II interface, the dominant contributors from the NOD1^CARD^ are R35, N36, and R101 (type-IIa), with RIP2^CARD^ residue I496 and additional contributions from the surrounding basic patch (Fig EK in S2 File). For the type-III interface, stabilizing energies primarily arise from the NOD1^CARD^ residues R35, T63, Q64, P65, K67, and R69 (type-IIIb) and RIP2^CARD^ residues K471, E472, and E475 (type-IIIa) (Fig EL in S2 File).

The residues identified across the NOD1^CARD^–RIP2^CARD^ heterodimeric interfaces closely align with previous experimental findings. At the top-down terminus, NOD1^CARD^ residues R69 (type-Ia), E53 (type-IIb/IIIa), and E56 (type-IIIa), together with RIP2^CARD^ residues D461 and Y474 (type-Ib) and R483 and R488 (type-IIIb), have been experimentally shown to be essential for NOD1–RIP2 association [22,24]. At the bottom-up terminus, NOD1^CARD^ residues D42 (type-Ib) and R69 (type-IIIb), along with RIP2^CARD^ residues R444, R488, and D492 (type-Ia) and E472, E475 (type-IIIa), have already been identified as critical for NOD1–RIP2 interaction and NF-κB signaling [22,24–26]. Taken together, the close agreement between prior experimental findings and our simulation data supports a model in which NOD1^CARD^ forms a short filament that is structurally compatible with RIP2^CARD^ filament at both termini. The additional residues identified here as potential contributors to homo- and heterodimeric interactions provide new mechanistic insights but will require further experimental validation to determine their functional significance.

### 2.7. Dynamic association of NOD2 tandem CARDs

**Fig 4:**
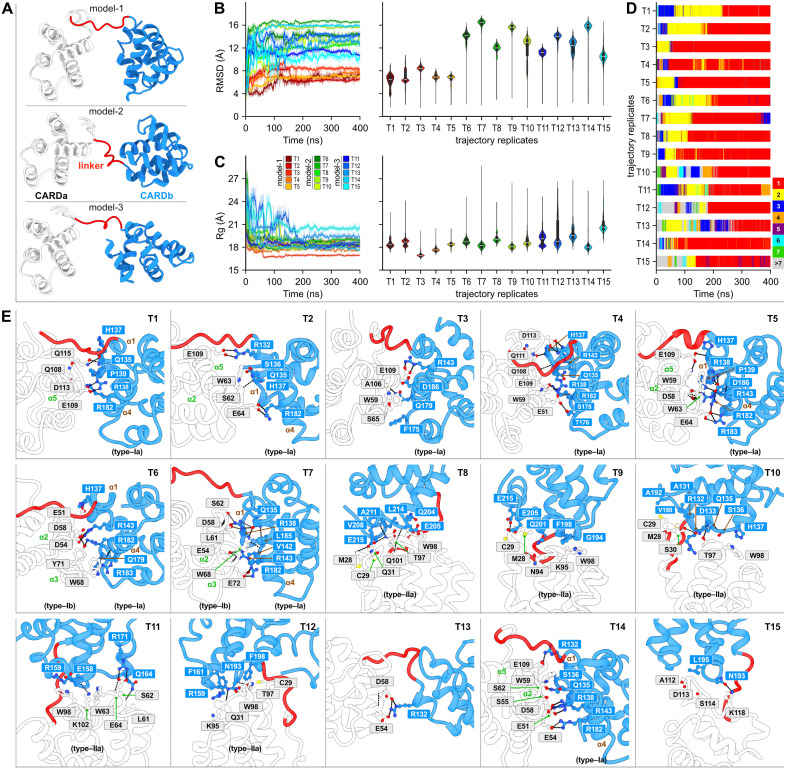
Dynamic association of NOD2 tandem CARDs. (A) Structural representation of NOD2 tandem CARDs showing CARDa, CARDb, and the inter–domain linker in gray, blue, and red cartoon, respectively. (B) Backbone RMSD and (C) Rg graphs of the three NOD2 tandem CARD models over 400 ns of MD simulations across 15 trajectories; traces for model-1, model-2, and model-3 are shown in red, green, and blue shades, with violin plots summarizing replicate-specific distributions. (D) RMSD-based clustering analysis over time, illustrating conformational state distributions along the trajectories; each color corresponds to a distinct structural cluster, with red denoting the dominant state. (E) Representative inter-CARD interaction profiles from selected trajectories (T1–T15). Key interacting residues between CARDa and CARDb are shown as ball-and-stick models and labeled, and inter-domain H-bonds are indicated in black dashed lines.

Over the past several years, multiple studies have investigated the structural and functional roles of the tandem CARDs of NOD2 in RIP2-mediated CARD–CARD interactions [21,24,33,46]. In our previous study, we integrated findings from the Rittinger group [46] along with predictions from AlphaFold2 [47] and RosettaFold [48] to identify three dynamically stable NOD2^CARDab^ models. However, when these models were docked onto the RIP2^CARD^ filament, it resulted in steric clashes between adjacent tandem CARD subunits [33], leaving unresolved questions about how the tandem CARD associates and, in particular, the critical role of CARDb in mediating NOD2–RIP2 interactions. To investigate this further, we generated three tandem CARD models (Fig 4A) and simulated each for 400 ns (a cumulative of 2 μs per model; Table A in S1 File). Backbone RMSD and Rg values converged within the first 50–150 ns (RMSD: ~ 6–16 Å; Rg: ~ 16–21 Å), indicating stable association of the tandem CARDs (Fig 4B and 4C). Interdomain interaction stability was further evaluated by computing H-bonds over time (Fig F in S2 File). Most trajectories exhibited consistent H-bonding patterns, with T4, T5, T7, and T14 showing ~7–9 H-bonds. Conversely, T9, T13, and T15 maintained weaker but persistent contacts, with 1–2 H-bonds.

To define the key interfaces governing tandem CARD association, we performed RMSD-based clustering for each trajectory and selected dominant cluster representatives for detailed interaction analysis (Fig 4D). The interaction analysis revealed a wide diversity of binding modes across the 15 trajectories (Fig 4E). Trajectories T1–T5 and T14 converged on variations of a shared arrangement in which the negatively charged CARDa surface (helices α2 and α5) engages with the CARDb type-Ia interface, consistent with earlier observations [33,46]. Trajectories T6 and T7 displayed the most common configuration, characterized by interactions between CARDa type-Ib and CARDb type-Ia interfaces, stabilized by an extensive H-bond network and in agreement with AlphaFold2 and RosettaFold predictions [33]. Additionally, distinct interaction modes emerged in trajectories T8–T12, in which CARDa uses its conserved type-IIa interface to interact with CARDb. Collectively, these results demonstrate that tandem CARD association is supported by interfacial plasticity, with multiple energetically viable binding associations across trajectories.

Given earlier findings that the NOD2^CARDb^ type-Ib interface can interact with RIP2^CARD^ type-Ia interface [24,33], we next tested whether any of the simulated tandem CARD conformations are structurally compatible with RIP2^CARD^ filament. To test this, we docked the representative coordinates from each dominant NOD2^CARDab^ cluster individually onto the RIP2^CARD^ filament and generated 15 models (F1–F15). Except for F1 and F15 models, all conformations produced substantial steric clashes either between adjacent tandem CARD subunits or with RIP2 CARDs, and none could be incorporated into the filament architecture (Fig G in S2 File). These clashes typically displaced CARDb from the filament axis, preventing the canonical helical alignment. In sum, these findings suggest that additional, as-yet-uncharacterized, tandem CARD conformations are likely required to stabilize the NOD2-RIP2 hetero–CARD filament.

### 2.8. NOD2 requires both CARDs for self-assembly and RIP2^CARD^ filament elongation

**Fig 5:**
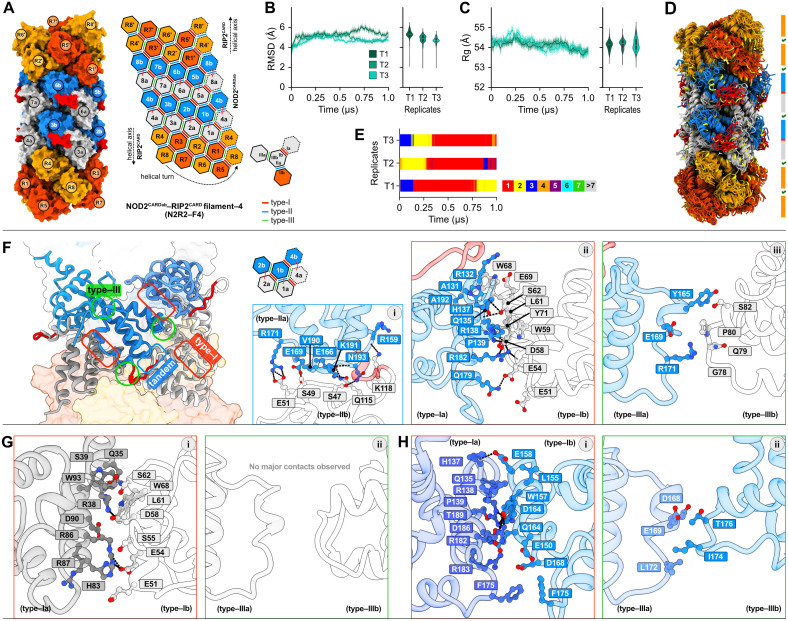
NOD2^CARDab^–RIP2^CARD^ interaction. (A) Structural and schematic overview of NOD2^CARDab^–RIP2^CARD^ filament model N2R2–F4. (B) Backbone RMSD and (C) Rg of the N2R2–F4 complex as a function of simulation time, reporting overall stability and compactness. (D) Conformational ensembles of the filament coordinates, sampled every 200 ns from three independent trajectories; CARD ring interfaces that satisfy canonical pairing are marked with green ticks. (E) RMSD-based clustering analysis of N2R2–F4 trajectories, with colors indicating distinct conformational clusters and red denoting the dominant state. (F) Intermolecular interactions within tandem NOD2^CARD^ rings: (i) detailed contacts between CARDa and CARDb at the tandem type-II interface, and at heterodimeric (ii) type-I and (iii) type-III interfaces. (G) Homodimeric interactions between CARDa subunits at (i) type-I and (ii) type-III interfaces. (H) Homodimeric interactions between CARDb subunits at (i) type-I and (ii) type-III interfaces, key residues are shown as ball-and-stick models and intermolecular H-bonds are indicated by black dotted lines.

NOD2 contains tandem CARDs similar to those of RLR proteins RIG-I and MDA5, whose tandem CARDs form filaments upon interaction with MAVS^CARD^ [35,36]. While NOD2^CARDa^ preferentially is known to interact with RIP2^CARD^ in a top-down orientation [30,31], RIG-I and MDA5 CARDb (CARD2) bind MAVS^CARD^ in a bottom-up configuration [35,36]. Despite these directional differences in their heterotypic interaction, tandem CARD association in both RIG-I and MDA5 is mediated predominantly through type-II interfaces. Motivated by these observations, we constructed a type-II NOD2^CARDab^ model (Fig H in S2 File) and subsequently built a NOD2^CARDab^–RIP2^CARD^ filament model (N2R2–F4), guided by known NOD2^CARDb^–RIP2^CARD^ interaction constraints [24,33]. The resulting filament model comprises two tiers of NOD2^CARDab^ rings flanked by two tiers of RIP2^CARD^ rings at termini (Fig 5A). The structural integrity of the N2R2–F4 model was assessed by performing three independent 1 μs MD simulations. As shown (in Fig 5B and 5C), the filament stabilized rapidly, with backbone RMSD (4.8–6.1 Å) and Rg (~54.2 Å) converging within the first ~100 ns, consistent with a compact, well-equilibrated complex. Conformational snapshots extracted every 200 ns (Fig 5D) showed strong structural convergence across trajectories, further supporting a stable and well-defined filament.

### 2.9. Heteromeric ring–ring interfaces stabilize NOD2–RIP2 filament

To further assess the stability of the hetero–CARD ring–ring interfaces, we quantified interfacial H-bonds across all trajectories and computed MM/PBSA binding free energies (Table F in S1 and Fig IA–IF in S2 Files). H-bond profiles revealed consistent yet dynamically fluctuating interactions at all three hetero–ring interfaces. The NOD2^CARDa^–NOD2^CARDb^ hetero–ring interface maintained a robust H-bond network (36.71 ± 2.75 bonds; Fig IA in S2 File), while the top-down NOD2^CARDa^–RIP2^CARD^ interface showed even higher counts (39.07 ± 6.10) (Fig IB in S2 File). The bottom-up NOD2^CARDb^–RIP2^CARD^ hetero–ring interface displayed somewhat fewer and more variable H-bonds (32.46 ± 6.12), consistent with a more dynamic yet still well-preserved interaction geometry (Fig IC in S2 File). MM/PBSA calculations revealed favorable binding free energies for all three ring–ring interfaces (Table F in S1 and Fig ID–IF in S2 Files). The NOD2^CARDa^–NOD2^CARDb^ hetero–ring interface was consistently strong across trajectories (ΔG_bind_ = −338.32 to −292.16 kcal/mol), driven by favorable ΔE_vdW_ and highly favorable ΔG_solv_ that compensate substantial electrostatic repulsion (Table I in S1 and Fig ID in S2 Files). The top-down NOD2^CARDa^–RIP2^CARD^ interface displayed similarly strong ΔG_bind_ (−340.42 to −255.44 kcal/mol) and was driven by ΔE_vdW_ and favorable ΔE_ele_ (Table I in S1 and Fig IE in S2 Files). In comparison, the bottom-up NOD2^CARDb^–RIP2^CARD^ interface showed weaker binding overall (ΔG_bind_ = −302.90 to −232.93 kcal/mol), reflecting a larger electrostatic penalty balanced by favorable ΔE_vdW_ and ΔG_solv_ contributions (Table I in S1 and Fig IF in S2 Files). Together, these results support a hierarchy of ring–ring stability within the N2R2–F4 filament: the NOD2^CARDa^–NOD2^CARDb^ and top-down NOD2^CARDa^–RIP2^CARD^ interfaces provide the strongest stabilization, whereas the bottom-up NOD2^CARDb^–RIP2^CARD^ interface, although stable, contributes weaker overall stabilization.

### 2.10. Interaction dynamics within tandem CARD rings

We assessed the structural integrity of the NOD2 tandem CARD rings by quantifying interfacial H-bonds and MM/PBSA binding free energies across all dimeric interfaces within the filament model (Fig 5F–5H, Fig J in S2 File). Seven distinct dimeric interfaces were identified: (1) the intramolecular tandem CARDa–CARDb interface (type-II); (2) two heterodimeric interfaces between NOD2 CARDa and CARDb (type-I/II); and (3) four homodimeric interfaces within the NOD2 rings, involving CARDa–CARDa and CARDb–CARDb contacts at type-I and type-III interfaces. For detailed interaction mapping and per-residue energy decomposition, 250 snapshots were extracted from the final 500 ns of N2R2–F4 (T2) trajectory (Fig 5E).

***Tandem CARD interaction is highly dynamic at the type-II interface***: The tandem CARDa–CARDb interface displayed a fluctuating H-bond network, with 3.08 ± 1.28 interfacial H-bonds across trajectories, consistent yet dynamically rearranging association (Fig JA in S2 File). Despite this variability, MM/PBSA analysis indicated moderately favorable ΔG_bind_ (−32.28 to −51.72 kcal/mol) (Table I in S1 and Fig JD in S2 Files). At the residue level, the interface is stabilized by a shifting ensemble of H-bond and vdW contacts between CARDa type-IIb and CARDb type-IIa interfaces (Fig 5Fi, Fig JG in S2 File). Per-residue energy decomposition highlights CARDa residues S47, S49, E51, Q115, and K118, together with CARDb residues R159, E169, R171, K191, and N193, as major contributors to interface stabilization (Fig JJ in S2 File). Together, these results indicate that the tandem interface remains intact but relatively weak and flexible, consistent with a hinge-like coupling that permits tandem CARD mobility during filament assembly.

***Heterodimeric type-I interface is the dominant stabilizing contact:*** Among heterodimeric interfaces, the type-I interface (CARDa type-Ib: CARDb type-Ia) exhibited the most persistent H-bonding network, maintaining 8.29 ± 1.09 H-bonds over time (Fig JB in S2 File), which was reflected by favorable ΔG_bind_ (−56.19 to −71.45 kcal/mol) (Table G in S1 File and Fig JE in S2 File). Interaction mapping revealed an extensive stabilizing network that combines multiple salt bridges and H-bonds (E54-R182, D58-R138/R182, S62-Q135/H137/R138, Y71-R138/R182/D186) with three key hydrophobic (W59-P139, W68-A131/A192) and several vdW contacts across the CARDa type-Ib and CARDb type-Ia surfaces (Fig 5Fii, and Fig JH in S2 File). In comparison, the heterodimeric type-III interface (CARDa type-IIIb: CARDb type-IIIa) was weak and transient, averaging fewer than one interfacial H-bond (Fig JC in S2 File) and exhibiting only modest ΔG_bind_ (−5.27 to −18.81 kcal/mol) (Table G in S1 and Fig JF in S2 Files). This interface is supported by a single transient H-bond and limited vdW contacts (Fig 5Fiii, Fig JI and JL in S2 File), indicating that it contributes only weakly compared with the dominant type-I interface.

***Homodimeric contacts within the NOD2 rings stabilize ring integrity**:* Within the CARDa ring, the type-I CARDa–CARDa interface maintained a stable H-bonding network (~5 H-bonds on average; Fig KA in S2 File) and a favorable ΔG_bind_ (−33.48 to −53.16 kcal/mol) (Table G in S1 File and Fig KE). This interface is stabilized by multiple salt-bridge and H-bond contacts (E51-R86/R87, E54-R86/R87, S55-H83/R86, D58-R38/R86), together with hydrophobic packing (W68-W93) and several vdW interactions (Fig 5Gi, and Fig KI in S2 File). Per-residue decomposition highlights R38, R86, R87 (type-Ia) and E51, E54, D58 (type-Ib) as key contributors (Fig KL in S2 File). In contrast, the type-III interface was highly transient with very few H-bonds over time (Fig KB in S2 File) and no persistent contact network (Fig 5Gii), resulting in only weakly favorable ΔG_bind_ (−1.28 to −6.79 kcal/mol) (Fig KF in S2 File). Within the CARDb ring, the type-I CARDb–CARDb interface exhibited greater stability than its CARDa counterpart, maintaining higher H-bond counts (5.77 ± 0.64) (Fig KC in S2 File) and stronger ΔG_bind_ (−43.17 to −65.49 kcal/mol) (Table G in S1 and Fig KG in S2 Files). This interface is supported by a dense network of salt-bridge/H-bonds (E150-R182, D154-R138/R182, W157-D186, D168-R183) and hydrophobic (L155-P139, F175-F175), and numerous vdW contacts (Fig 5Hi, and Fig KJ in S2 File). Per-residue decomposition identifies E150, D154, W157, E158, Q164, D168 (type-Ib), together with R138, R182, R183 (type-Ia) as principal contributors. In contast, the type-III interface remained weaker and more variable, exhibiting fewer H-bonds (Fig KD in S2 File), only modest ΔG_bind_ (−15.17 to −21.33 kcal/mol) (Table G in S1 and Fig KH in S2 Files), and only bare interfacial contacts (Fig 5Hii, and Fig KK and OK in S2 File). Overall, these data indicate that tandem CARD ring integrity is driven primarily by type-I interfaces, both heterodimeric and homodimeric, whereas the type-III contacts provide additional flexibility and contribute weakly to stabilization.

### 2.11. Interactions between CARDa and CARDb at the heterodimeric ring–ring interface

**Fig 6:**
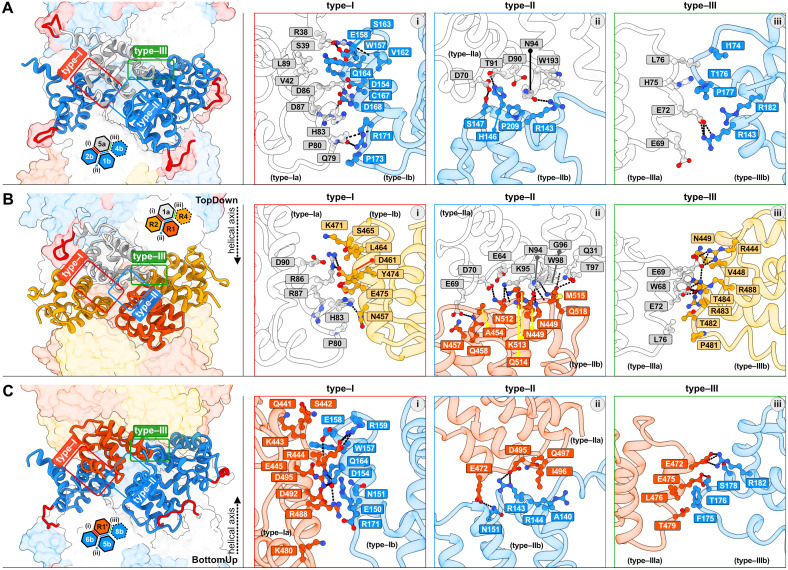
Hetero–CARD interaction modes at NOD2–NOD2 and NOD2–RIP2 ring–ring interfaces. (A) Representative contacts between NOD2^CARDa^ and NOD2^CARDb^ from vertically adjacent tandem CARD rings. (B) Interactions between NOD2^CARDa^ and RIP2^CARD^ at the top-down terminus. (C) Interactions between NOD2^CARDb^ and RIP2^CARD^ at the bottom-up terminus. In each panel (A–C), the three canonical interface modes are boxed and color-coded: type-I (red, i), type-II (blue, ii), and type-III (green, iii). Interacting residues are labeled and depicted as sticks.

To examine hetero–CARD stability across the three canonical interfaces, between the two NOD2^CARDab^ rings and with RIP2^CARD^ at both the top-down and bottom-up termini, we quantified interfacial H-bonds, computed MM/PBSA binding free energies, mapped interaction profiles, and carried out per-residue energy decomposition (Fig 6A–C, Table H in S1 File, and Fig L–O in S2 File). At the CARDa–CARDb type-I interface, H-bond analysis showed a stable, moderately dense network that persisted across the trajectories (4.40 ± 1.88; Fig LA in S2 File). This is supported by favorable ΔG_bind_ (−33.83 to −57.42 kcal/mol) (Table H in S1 and Fig LD in S2 Files), indicating that the type-I interface stabilizes CARDa–CARDb association. The type-II interface showed a comparable, though slightly more variable, H-bonding pattern (3.56 ± 0.71; Fig LB in S2 File) and similarly favorable ΔG_bind_ (−39.65 to −61.26 kcal/mol) (Table H in S1 and Fig LE in S2 Files), suggesting that it also contributes strongly to heterodimer stability. In contrast, the type-III interface formed a similar number of H-bonds to type-II (3.00 ± 0.90; Fig LC in S2 File) but weaker ΔG_bind_ (−20.3 to −26.2 kcal/mol) (Table H in S1 and Fig LD in S2 Files), indicating a less energetically dominant CARDa–CARDb association.

Type-I interface revealed a strong interaction comprising multiple H-bonds (R38-W157/V162, Q79-R171, S82-D154, R86-D154, R87-D168), hydrophobic (V42/L89-W157), and several vdW contacts between the CARDa type-Ia and CARDb type-Ib interfaces (Fig 6Ai, and Fig LG in S2 File). Per-residue energy decomposition identified CARDa residues R38, Q79, S82, R86, R87, together with CARDb residues D154, W157, R171 as major contributors to the binding (Fig LJ in S2 File). At the type-II interface, interactions predominantly occur between the CARDa type-IIa and CARDb type-IIb surface patches and include three to four H-bonds (D70-S147, D90/T91-H146, N94-R143/H146) accompanied by multiple vdW contacts (Fig 6Aii, and Fig LH in S2 File). Per-residue decomposition at this interface highlighted D70, D90, W93, N94 (CARDa type-IIa), along with R143, R144, H146, S147 (CARDb type-IIb), as critical stabilizing residues (Fig LK in S2 File). The interaction at the type-III interface is dominated by electrostatic/H-bond pairs (E69/E72-R143, E72-R182), complemented by a single hydrophobic contact (L76-I174) and several vdW contacts (Fig 6Aiii, and Fig LI in S2 File). Here, E69 and E72 (CARDa type-IIIa) and R143 and R182 (CARDb type-IIIb) emerge as principal contributors to the NOD2–NOD2 hetero–CARD interaction (Fig LL in S2 File). In sum, these data define a clear hierarchy of CARDa–CARDb heterodimeric contacts: type-I and type-II interfaces provide the dominant stabilizing interactions, whereas type-III interfaces contribute a weaker, secondary stabilizing component.

### 2.12. NOD2^CARDs^ interact with RIP2^CARD^ at both termini

Like the NOD1–RIP2, the NOD2–RIP2 hetero–CARD interaction has also been extensively studied. Wagner et al. showed that charge-reversal mutations of NOD2^CARDa^ residues E69, D70, and E71 disrupt RIP2^CARD^ binding [23]. Subsequently, Fridh and Rittinger identified NOD2^CARDa^ residues R38, E69, and R86 together with RIP2^CARD^ residues D461, E472, D473, E475, and D492, as critical for NOD2–RIP2 association [24]. A potential type-II binding mode involving the RIP2^CARD^ type-IIb residue T452 and NOD2^CARDa^ has also been proposed [31]. Based on these findings, our previous work suggested that NOD2–RIP2 association may engage both type-I and type-II interaction modes, mediated by NOD2^CARDa^ type-Ia/IIa interfaces and RIP2^CARD^ type-Ib/IIb interfaces [33]. More recently, two cryo-EM studies have indicated that the top-down interface is crucial for NOD2–RIP2 CARD–CARD filament formation [30,31]. Despite these advances, residue-level contributions across the three canonical interfaces (type-I, II, and III) remain only partially resolved. Moreover, because NOD2 harbors tandem CARDs, the specific role of CARDb in RIP2 recruitment and filament growth remains unclear [30,31,33]. Several residues implicated in NOD2^CARDa^–RIP2^CARD^ binding also map to alternative interface surfaces [23,24], raising the possibility of multiple interaction modes.

### 2.13. Interaction between NOD2^CARDa^ and RIP2^CARD^ at top-down terminus

At the top-down type-I interface, a modest but persistent H-bonding network is maintained, with a trajectory-averaged H-bond count of 3.44 ± 0.72 (Fig MA in S2 File), yielding moderately favorable ΔG_bind_ (−26.28 to −32.87 kcal/mol) (Table H in S1 and Fig MD in S2 Files). The type-II interface exhibits slightly stronger H-bonding (4.46 ± 1.53 H-bonds; Fig MB in S2 File) and the strongest ΔG_bind_ (−46.68 to −51.66 kcal/mol) among the three top-down modes (Table H in S1 and Fig ME in S2 Files). In comparison, the type-III interface shows the highest H-bond counts (5.26 ± 1.10 H-bonds; Fig MC in S2 File), but weaker and more variable ΔG_bind_ (−15.69 to −29.57 kcal/mol) (Table H in S1 and Fig MF in S2 Files), with stabilization driven primarily by attractive electrostatics. In summary, these results indicate that the type-II top-down interface is the dominant stabilizing mode, with type-I providing intermediate support and type-III remaining energetically weaker despite its higher H-bond occupancy.

To define the molecular determinants of these interfaces, we mapped interaction networks and performed per-residue energy decomposition (Fig 6B, and Fig MG–ML in S2 File). Across the canonical interfaces, the interaction stability is governed by H-bond/electrostatic contacts supported by vdW packing (Fig MG–MI in S2 File). The type-I interface showed five to six dynamic polar contacts (H83-N457/Y474, R86-D461/S465, R87-E475, D90-K471) and several vdW contacts between the NOD2^CARDa^ type-Ia and RIP2^CARD^ type-Ib surface patches (Fig 6Bi, and Fig MG in S2 File). Per-residue energetic decomposition highlights NOD2^CARDa^ residues R38, H83, R86, and R87 and RIP2^CARD^ residues N457, Q485, D461, R466, Y474, and K486 as key contributors (Fig MJ in S2 File). The type-II interface features a broader interaction network of H-bonds/electrostatic contacts (E64-K513, E69-N457/Q458, D70-N512, N94-M515, K95-N512/Q514/Q518, T97-Q518, W98-K513/Q518), again supported by extensive vdW packing (Fig 6Bii, and Fig MH in S2 File). Here, the NOD2^CARDa^ (type-IIa) residues E64, E69, D72, N94, K95, and W98, and RIP2^CARD^ (type-IIb) residues N457, Q458, R483, K510, N512, K513, Q514, M515, and Q518 contribute favorably to binding (Fig MK in S2 File). The type-III interface is stabilized by three principal H-bonds linking E69/E72 (NOD2^CARDa^ type-IIIa) with R444, T484, and R488 (RIP2^CARD^ type-IIIb), and these residues also emerged as favorable energetic hotspots (Fig 6Biii, and Fig MI and ML in S2 File).

Importantly, the residues identified here align closely with prior mutational and biochemical data. NOD2^CARDa^ residues R38 and R86 (type-Ia) and E69/E72 (type-IIIa), together with RIP2^CARD^ residues D461 and Y474 (type-Ib), R483/R488 (type-IIIb), and the previously proposed T452-linked type-II mode, correspond to sites shown experimentally to regulate NOD2–RIP2 association [23,24,31]. This convergence of simulations and experiments supports a multi-interface, top-down interaction between NOD2^CARDa^–RIP2^CARD^, while the additional hotspots identified here provide testable predictions for future experimental validation.

### 2.14. Role of NOD2^CARDb^ in NOD2–RIP2 Interaction (bottom-up interface interaction)

Previous studies have suggested that NOD2^CARDb^ plays a key role in RIP2-mediated NF-κB signaling and hetero–CARD interaction [21,33]. We therefore quantified H-bonds and MM/PBSA energetics across the three canonical bottom-up interfaces, complemented by interaction mapping and per-residue energy decomposition (Table H in S1 File and Fig NA–NL in S2 File). Among the bottom-up interfaces, the type-I displayed the most persistent H-bonding (8.01 ± 1.22 H-bonds; Fig NA in S2 File) and the most favorable ΔG_bind_ (−47.11 to −51.02 kcal/mol) (Table H in S1 File and Fig ND). In contrast, the type-II and type-III interfaces formed weaker and more variable contacts: type-II showed fewer H-bonds (1.97 ± 1.47 H-bonds; Fig NB in S2 File) with moderately favorable ΔG_bind_ (−15.25 to −31.63 kcal/mol) (Table H in S1 File and Fig NE), whereas type-III showed slightly higher H-bond counts (2.87 ± 0.29) (Fig NC in S2 File), and modest ΔG_bind_ (−21.90 to −22.71 kcal/mol) (Table H in S1 File and Fig NF).

The dynamic interaction map indicated that the bottom-up NOD2^CARDb^–RIP2^CARD^ interfaces are stabilized by coordinated H-bonds and vdW networks (Fig 6C, and Fig NG–NI in S2 File). At the type-I interface, NOD2^CARDb^ (type-Ib) interacts with RIP2^CARD^ (type-Ia) through multiple H-bonds (D154-R444/R488, E158-R444/E445, R159-E445, Q164-D492), electrostatic pairs (E150-K480/R488, E158-K443, R171-D492), and several vdW contacts (Fig 6Ci, and Fig NG in S2 File). Per-residue energy contribution identifies NOD2^CARDb^ residues E150, D154, R159, and Q164, and RIP2^CARD^ residues R444, K480, and R488 as major contributors to the interaction (Fig NJ in S2 File). The type-II interface is supported by a smaller polar network comprising three H-bonds (R143-D495, R144-Q497, N151-E472) with limited vdW contacts (Fig 6Cii, and Fig NH in S2 File), with NOD2^CARDb^ residues R143 and R144 (type-IIb) and RIP2^CARD^ residues R466, K471, D492, and D495 (type-IIa) contributing most strongly to binding (Fig NK in S2 File). The type-III interface forms a comparable H-bonding network (T176-E475, S178/R182-E472) supplemented by hydrophobic and vdW contacts (Fig 6Ciii, and Fig NI in S2 File); here, energetic hotspots include NOD2^CARDb^ residues R143, T176, S178, and R182, and RIP2^CARD^ residues E472, D473, and E475 (Fig NL in S2 File). Together, these data indicate that type-I stability arises from a dense, multi-contact network. In contrast, type-II and type-III interfaces are maintained by smaller, more localized interaction clusters that provide weaker but dynamic secondary support.

The key residues identified for NOD2^CARDb^ and RIP2^CARD^ are consistent with previous reports [22,24]. RIP2^CARD^ residues R444 and R488 (type-Ia), previously shown to interact with NOD1^CARD^ [22], and residues D492 (type-Ia), E472, E475 (type-IIa/IIIa), implicated in NOD2–RIP2 binding and NF-κB signaling [24], are all recovered as energetic hotspots, and the type-I NOD2^CARDb^–RIP2^CARD^ contacts agree well with our earlier predictions [33]. Overall, these results support a model in which the NOD2^CARDb^–RIP2^CARD^ interaction at the bottom-up terminus is stabilized primarily through the dominant type-I interface. At the same time, the type-II and type-III contacts act as weaker, auxiliary interactions. The resulting NOD2^CARDb^–RIP2^CARD^ bottom-up configuration closely resembles that observed in the NOD1^CARD^–RIP2^CARD^ filament (Fig 3H) and in RIG-I/MDA5^CARD^–MAVS^CARD^ filaments, suggesting a conserved architectural principle for CARD-driven filament elongation [35,36].

### 2.15. *In silico* mutagenesis validates interface specificity

**Fig 7:**
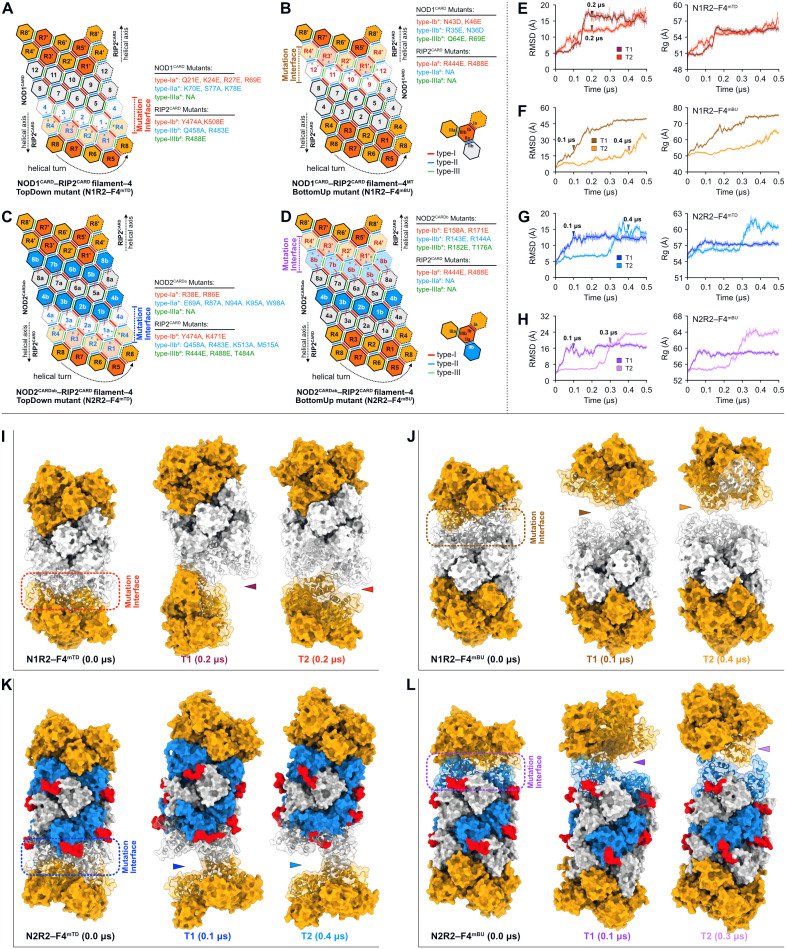
*In silico* mutagenesis reveals interface-specific destabilization of NOD1–RIP2 and NOD2–RIP2 heterotypic CARD filaments. (A–D) Schematic representations of mutant filament models highlighting charge-reversal and alanine substitutions at inter-ring interfaces: (A) N1R2–F4^mTD^ (top-down), (B) N1R2–F4^mBU^ (bottom-up), (C) N2R2–F4^mTD^ (top-down), and (D) N2R2–F4^mBU^ (bottom-up). Mutated residues are indicated and color-coded by interface type: type-I (red), type-II (blue), and type-III (green). (E–H) Backbone RMSD (left) and radius of gyration (Rg; right) over 0.5 μs MD simulations for two independent trajectories (T1 and T2): (E) N1R2–F4^mTD^, (F) N1R2–F4^mBU^, (G) N2R2–F4^mTD^, and (H) N2R2–F4^mBU^. All mutant systems exhibit progressive structural divergence and loss of compactness relative to wild-type assemblies. (I–L) Representative snapshots from independent trajectories at indicated time points showing progressive dissociation of RIP2 CARD rings from NOD1/2 filament scaffolds: (I) N1R2–F4^mTD^, (J) N1R2–F4^mBU^, (K) N2R2–F4^mTD^, and (L) N2R2–F4^mBU^. NOD1^CARD^ is shown in light gray, NOD2^CARDa^ in gray, NOD2^CARDb^ in blue, the NOD2 linker in red, and RIP2^CARD^ in orange. Mutated CARD subunits are displayed in cartoon-transparent surface representation.

To rigorously assess the specificity of the proposed NOD1/2–RIP2 CARD interfaces and exclude the possibility that filament stability arises from nonspecific or force-field–driven interactions, we performed targeted *in silico* mutagenesis. Guided by literature-defined hotspot residues [22–26,29–31,33] and our interface mapping, we introduced charge-reversal and alanine-substitution mutations at both top-down and bottom-up hetero–CARD interfaces (Fig 7A–D, and Table I in S1 File). For the NOD1–RIP2 system, two mutant filament models were generated: N1R2–F4^mTD^ (top-down; Fig 7A) and N1R2–F4^mBU^ (bottom-up; Fig 7B). Analogous mutants were constructed for the NOD2–RIP2 system: N2R2–F4^mTD^ (top-down; Fig 7C) and N2R2–F4^mBU^ (bottom-up; Fig 7D). All mutant systems were subjected to a 0.5 μs production run in duplicate under conditions identical to those used for the wild-type systems.

All mutant systems exhibited rapid and reproducible disruption of filament architecture. In NOD1–RIP2 mutant systems, both RMSD and Rg profiles showed significant divergence from the stable wild-type trajectories. N1R2–F4^mTD^ showed a continuous rise in RMSD (~15–20 Å), while N1R2–F4^mBU^ exhibited similarly pronounced destabilization (~30–45 Å), accompanied by a significant increase in Rg (Fig 7E–F). Similarly, in NOD2–RIP2 mutant systems, both N2R2–F4^mTD^ and N2R2–F4^mBU^ showed significant increases in RMSD and Rg (Fig 7G–H), consistent with a loss of filament compactness. Complementary analyses further supported these findings, with increased SASA, elevated interfacial RMSD, and reduced interfacial H-bonds collectively indicating progressive weakening of inter-subunit contacts (Fig O in S2 File). Structural snapshots at distinct time points showed that mutations at either interface (top-down or bottom-up) led to progressive dissociation of RIP2 CARD rings from the filament ends, with clear separation events observable early in the trajectories (0.1–0.4 μs) (Fig 7I–L). Collectively, these results provide strong supporting evidence that (1) the identified interfaces are specific rather than generic sticky contacts, (2) the key residues identified through interaction mapping and energy decomposition are indeed critical for interface integrity, and (3) our computational workflow can successfully discriminate between viable and disrupted filament architectures.

## 3. Conclusion

Upon recognizing specific peptidoglycan motifs, NOD1 and NOD2 undergo ATP-dependent oligomerization, recruit RIP2 via CARD–CARD interactions, and activate the NF-κB signaling cascade. Although multiple studies have examined the interfaces and residues that govern NOD1/2–RIP2 interactions, the molecular details remain unclear [27]. Recent cryo-EM studies of RIP2^CARD^ filaments have highlighted that structural heterogeneity has hindered the determination of high-resolution hetero–CARD filament structures [30,31]. This challenge is likely amplified by the highly conserved CARD fold; even in genuine hetero–CARD assemblies, NOD1-, NOD2-, and RIP2-derived densities are expected to appear very similar. In such cases, near-isomorphous subunits are difficult to distinguish in cryo-EM reconstructions, and subtle compositional differences may be averaged out, masking receptor-specific interfaces and filament polarity. These limitations motivated us to investigate, at atomic detail, the critical interfaces and residues that stabilize NOD1/2–RIP2 CARD–CARD filaments.

In this study, we dissected both conserved and receptor-specific interaction modalities between the CARDs of NOD1/NOD2 and RIP2. In the NOD1–RIP2 hetero–CARD filament model, all six NOD1^CARD^ interfaces participate in interactions at both the homodimeric (NOD1–NOD1) and heterodimeric (NOD1–RIP2) interface. These results are consistent with previous proposals that NOD1^CARD^ is structurally compatible with a short-filament scaffold configuration that can accommodate both top-down and bottom-up association of the RIP2^CARD^ filament [30]. Our simulations further indicate that the bottom-up configuration is structurally viable. Although the feasibility of bottom-up RIP2^CARD^ filament conformation on NOD1^CARD^ scaffold could be questioned, the observed hetero–CARD interactions at type-I and type-III interfaces (Fig 3H) and earlier mutagenesis data [22,24], strongly support the viability of both configurations. In contrast, NOD2 exhibits a more asymmetric structural mechanism. For the NOD2–RIP2 hetero–CARD filament, our simulations indicate that NOD2 tandem CARDs can adopt a short filament scaffold configuration that is structurally compatible with RIP2^CARD^. In the top-down configuration, interactions are mediated by the CARDa ring, whereas at the bottom-up terminus, CARDb ring interfaces provide the structural basis for RIP2 association, highlighting distinct functional roles for the two domains. The targeted *in silico* mutagenesis further demonstrates that these interfaces are highly specific and critically dependent on defined hotspot residues, as their disruption led to rapid filament destabilization. These findings distinguish physiologically relevant interactions from nonspecific contacts and validate the proposed hetero–CARD interfaces and filament models.

This work provides a unified structural framework for NOD1/2–RIP2 hetero–CARD interactions that are in close agreement with previous experimental and computational studies [22–26,29–31,33]. For the NOD2–RIP2 system, the N2R2–F4 filament model was constructed under the hypothesis that NOD2 tandem CARDs adopt a type-II intramolecular arrangement analogous to RIG-I/MDA5–MAVS filament structures [35,36]. While our results show that this arrangement is geometrically compatible with the RIP2^CARD^ filament and remains stable over the simulation timescale, its biological relevance remains to be established. Alternative arrangements that satisfy the geometric constraints of the filament may also exist, and experimental validation will be required to distinguish among these possibilities.

Our findings support the model in which conserved CARD interfaces enable filament stability, while receptor-specific features, particularly the conformational plasticity of NOD2 tandem CARDs and the central role of CARDb, govern adaptor recruitment and filament configuration. We emphasize that these simulations evaluate the structural compatibility and persistence of pre-constructed interfaces rather than defining a kinetic assembly pathway; accordingly, the conclusions reflect the relative stability of the modeled filament complexes rather than a resolved temporal mechanism. Overall, this study provides mechanistic insight into NOD1/2–RIP2 CARD–CARD interactions, defining the structural basis of filament assembly and receptor-specific mechanisms that distinguish NOD1 and NOD2.

## 4. Methods

### 4.1. Modeling of NOD1/2-RIP2 CARD-CARD filaments

Experimental structures of NOD1^CARD^ (PDB ID: 2DBD; model 1 from the NMR ensemble) and RIP2^CARD^ (2N7Z [49], 5YRN [30], 6GGS [31]) were obtained from the Protein Data Bank (PDB) (https://www.rcsb.org). The 3D models of NOD2 tandem CARDs were generated as described previously [33]. To generate NOD1/2–RIP2 CARD–CARD filament models, we docked CARD subunits into the cryo-EM map of RIP2^CARD^ filament (EMD-6842) [30] using UCSF ChimeraX [50], guided by published NOD1/2–RIP2 interaction data [22–26,30,31]. The subunit composition of each model was determined by the helical symmetry of the cryo-EM structure, in which four CARD subunits constitute one helical turn. Eleven filament models were generated (Table A in S1 File), and the details of the modeling are provided in the Results and Discussion section.

### 4.2. MD simulation of NOD1/2-RIP2 filament models and NOD2 tandem CARDs

To gain insight into the structure and dynamic properties of the NOD1/2–RIP2 filament and NOD2 tandem CARD models, MD simulations were performed using the GROMACS simulation suite [51] and the Amber ff99SB*-ILDN-Q force field [52,53], which has been validated for balanced treatment of secondary structure propensities and is suitable for simulating flexible linker regions. A buffer distance of 10–12 Å was maintained between the protein surfaces and the box edges to allow sufficient movement of macromolecule(s) and to prevent interactions with periodic images. Each simulation system was solvated with TIP3P water in individual cubic/triclinic boxes and neutralized with 0.15 M Na^+^ and Cl^-^ ions. Periodic boundary conditions were applied to minimize the boundary-induced artifacts. Prior to production runs, each system was subjected to energy minimization using the steepest descent algorithm [54] until a convergence threshold of 1000 kJ.mol^-1^.nm^-1^ was reached, thereby resolving steric clashes and reducing high-energy interactions by optimizing molecular geometries. The energy-minimized systems were then equilibrated in two sequential steps: an NVT ensemble (constant number of particles, volume, and temperature) for 1 ns, followed by an NPT ensemble (constant number of particles, pressure, and temperature) for 5 ns. During equilibration and production, the system temperature was maintained at 300 K using the velocity-rescale (V-rescale) thermostat [55], with a coupling constant (τ_T_) of 0.1 ps. The pressure was maintained at 1 bar with the Parrinello-Rahman barostat method [56]. Particle-Mesh Ewald (PME) summation method was employed to compute long-range electrostatic interactions [57]. The LINCS algorithm was used to constrain all covalent bonds [58], and a time step of 2 fs was employed for data collection. All subunits, including those at filament termini, were free to move without any position restraints during production runs. Final production runs for the NOD2 tandem CARD and hetero–CARD filament models were performed with multiple replicates, each spanning 200 ns to 1 μs (Table A in S1 File).

### 4.3. Analysis of MD trajectories

Following the generation of the MD trajectories, comprehensive analyses were performed using the built-in modules of GROMACS. Structural stability was evaluated by calculating the root-mean-square deviation (RMSD) and the radius of gyration (Rg) over time using the commands ‘*gmx rms*’ and ‘*gmx gyrate*’, respectively. To analyze the conformational states explored during the simulation, we performed RMSD-based clustering with ‘*gmx cluster*’. Principal component analysis (PCA) of the mainchain atoms was performed with ‘*gmx covar*’ and ‘*gmx anaeig*’. While the solvent-accessible surface area (SASA) was assessed using ‘*gmx sasa*’, interfacial hydrogen bonds (H-bonds) were determined using ‘*gmx hbond*’.

To map dynamic interactions at CARD–CARD interfaces, we developed a custom Python script and used MDAnalysis [59] to parse trajectories and detect interactions. For each homo- and heterodimeric interface, we extracted 250 evenly spaced snapshots from the final 500 ns of each selected trajectory and computed residue-residue contact frequencies to identify persistent interfacial contacts that likely stabilize CARD–CARD filament assembly. ChimeraX [50] was used for visualizing 3D models and residue-level interactions. Binding free energy and per-residue contributions were estimated using the Molecular Mechanics Poisson-Boltzmann Surface Area (MM/PBSA) method implemented in the gmx_MMPBSA tool [60]. For these calculations, 200 snapshots (from 200 ns trajectory) or 250 snapshots (from 1 μs production run) were evenly sampled from the final half of each trajectory. MM/PBSA-derived binding energies were used as comparative metrics to qualitatively rank relative interface binding affinities across models and interfaces. These values do not represent absolute thermodynamic binding free energies, as the MM/PBSA method typically omits configurational entropy contributions and is sensitive to parameter choices. 2D Plots and dynamic interaction maps were generated with Grace 5.1.21 (http://plasma-gate.weizmann.ac.il/Grace/) and Matplotlib [61].

## Supporting information

S1 File Supporting Tables A–I. (PDF)

S2 File Supporting Figures A–O. (PDF)
