# Supplementary material for: Structural and dynamic basis of NOD2 tandem CARD association and NOD1/2–RIP2 signaling complexes
Source: PLoS Comput Biol. 2026 May 29;22(5):e1014311. doi: 10.1371/journal.pcbi.1014311 (PMC13249360; doi:10.1371/journal.pcbi.1014311)
Supplement: S1 File — (PDF) [file pcbi.1014311.s001.pdf]

Supporting Information | S1 File (Supporting Tables)

Structural and Dynamic Basis of NOD2 Tandem CARD Association and NOD1/2–RIP2 Signaling Complexes

Jitendra Maharana[1,2,3,*], Aritra Bej[4,5], Debasish Biswal[6,7,8], Debashis Panda[9], and Arjun Sharma[10,*]

[1]Institute of Biological Chemistry, Academia Sinica, Taipei 11529, Taiwan. [2]Taiwan International Graduate Program (TIGP), Chemical Biology and Molecular Biophysics (CBMB), Academia Sinica, Taipei, 11529, Taiwan. [3]Institute of Bioinformatics and Structural Biology, College of Life Sciences and Medicine, National Tsing Hua University, Hsinchu 300, Taiwan. [4]Department of Chemistry and [5]Department of Pharmacology, University of California, Davis, CA, 95616, USA. [6]Institute of Molecular Biology, Academia Sinica, Taipei 11529, Taiwan. [7]Taiwan International Graduate Program (TIGP) - Interdisciplinary Neuroscience (INS), Academia Sinica, 11529, Taiwan. [8]College of Life Science, National Taiwan University, Taipei, 10617, Taiwan. [9]DBT-APSCS&T, Centre of Excellence for Bioresources and Sustainable Development, Kimin, Arunachal Pradesh 791121, India. [10]Department of Chemistry and Biochemistry, Purdue University Fort Wayne, Fort Wayne, Indiana 46805, USA.

*Correspondence: jitued@gmail.com (J.M.); arjun.sharma@pfw.edu (A.S.)

Table A. Summary of MD simulation systems, including simulation length, replicate runs and figure reference.

Simulation Systems	Total No. of Atoms	Time | No. of Runs	Figure Ref.
NOD1/2 CARD(a) – RIP2 CARD filament models			
NOD1^{CARD} (×4)–RIP2^{CARD} (×8) filament 1 (N1R2–F1)	1,66,927	200 ns | ×3	Fig 1A
NOD2^{CARDa} (×4)–RIP2^{CARD} (×8) filament 1 (N2aR2–F1)	1,63,113	200 ns | ×3	
NOD1^{CARD} (×4)–RIP2^{CARD} (×8) filament 2 (N1R2–F2)	1,61,150	200 ns | ×3	
NOD2^{CARDa} (×4)–RIP2^{CARD} (×8) filament 2 (N2aR2–F2)	1,72,324	200 ns | ×3	
NOD1^{CARD} (×8)–RIP2^{CARD} (×4) filament 3 (N1R2–F3)	1,67,576	200 ns | ×3	Fig 2A
NOD2^{CARDa} (×8)–RIP2^{CARD} (×4) filament 3 (N2aR2–F3)	1,64,485	200 ns | ×3	
NOD1^{CARD}/NOD2^{CARDab}–RIP2^{CARD} long filament models			
NOD1^{CARD} (×12)–RIP2^{CARD} (×16) filament 4 (N1R2–F4)	2,06,444	1 μs | ×3	Fig 3A
NOD2^{CARDab} (×8)–RIP2^{CARD} (×16) filament 4 (N2R2–F4)	2,21,417	1 μs | ×3	Fig 5A
NOD1^{CARD}/NOD2^{CARDab}–RIP2^{CARD} long filament mutant models			
NOD1^{CARD} (×12)–RIP2^{CARD} (×16) filament 4 top-down mutant (N1R2–F4^{mTD})	2,06,541	0.5 μs | ×2	Fig 7A
NOD1^{CARD} (×12)–RIP2^{CARD} (×16) filament 4 bottom-up mutant (N1R2–F4^{mBU})	2,06,617	0.5 μs | ×2	Fig 7B
NOD2^{CARDab} (×8)–RIP2^{CARD} (×16) filament 4 top-down mutant (N2R2–F4^{mTD})	2,19,692	0.5 μs | ×2	Fig 7C
NOD2^{CARDab} (×8)–RIP2^{CARD} (×16) filament 4 bottom-up mutant (N2R2–F4^{mBU})	2,20,653	0.5 μs | ×2	Fig 7D
NOD2 tandem CARDs			
Model-1	59,007	400 ns | ×5	Fig 4A
Model-2	1,02,961	400 ns | ×5	
Model-3	91,752	400 ns | ×5	

Table B. MM/PBSA energy components (kcal/mol) for NOD1^{CARD}–RIP2^{CARD} and NOD2^{CARDa}–RIP2^{CARD} heteromeric ring–ring interfaces.

Interface | Trajectory		ΔE_{vdW}	ΔE_{ele}	ΔG_{solvp}	ΔG_{solvnp}	ΔE_{gas}	ΔG_{solv}	ΔG_{bind}
N1R2-F1 – NOD1^{CARD}–RIP2^{CARD} heteromeric ring interface								
Top-down (Fig AD)	T1	-292.81 ± 12.70	-131.80 ± 20.53	147.01 ± 17.79	-33.02 ± 0.76	-424.61 ± 23.98	113.99 ± 17.48	-310.63 ± 12.45
	T2	-296.55 ± 13.65	-138.89 ± 22.30	156.95 ± 18.67	-33.29 ± 0.88	-435.45 ± 25.57	123.66 ± 18.28	-311.79 ± 13.23
	T3	-318.61 ± 14.01	-144.98 ± 25.19	160.19 ± 20.94	-34.73 ± 1.33	-463.60 ± 27.93	125.46 ± 20.37	-338.13 ± 13.86
N2aR2-F1 – NOD2^{CARDa}–RIP2^{CARD} heteromeric ring interface								
Top-down (Fig AD)	T1	-274.44 ± 14.07	-62.01 ± 21.37	79.56 ± 17.73	-31.71 ± 0.93	-336.45 ± 24.48	47.85 ± 17.26	-288.61 ± 12.28
	T2	-279.13 ± 14.79	-7.51 ± 26.89	38.68 ± 25.15	-33.13 ± 1.11	-286.65 ± 35.46	5.55 ± 24.45	-281.10 ± 14.51
	T3	-243.78 ± 25.79	-22.27 ± 36.63	39.05 ± 33.88	-30.82 ± 2.13	-266.05 ± 55.26	8.23 ± 32.14	-257.82 ± 28.08
N1R2-F2 – NOD1^{CARD}–RIP2^{CARD} heteromeric ring interface								
Bottom-up (Fig AD)	T1	-196.65 ± 11.55	163.94 ± 20.69	-135.77 ± 17.46	-23.15 ± 1.13	-32.71 ± 23.56	-158.92 ± 17.41	-191.63 ± 10.83
	T2	-248.48 ± 12.33	150.85 ± 17.84	-118.29 ± 15.10	-30.30 ± 1.01	-97.63 ± 20.73	-148.59 ± 14.74	-246.22 ± 10.56
	T3	-225.60 ± 11.63	137.59 ± 16.41	-106.21 ± 14.23	-27.75 ± 1.09	-88.01 ± 22.18	-133.96 ± 13.64	-221.97 ± 11.77
N2aR2-F2 – NOD1^{CARDa}–RIP2^{CARD} heteromeric ring interface								
Bottom-up (Fig AD)	T1	-115.34 ± 21.01	502.13 ± 21.08	-456.18 ± 19.33	-14.78 ± 3.10	386.78 ± 33.40	-470.96 ± 18.03	-84.18 ± 21.52
	T2	-144.78 ± 24.43	518.93 ± 25.83	-462.79 ± 24.45	-17.64 ± 3.55	374.14 ± 37.76	-480.44 ± 22.83	-106.29 ± 20.98
	T3	-102.71 ± 16.40	513.60 ± 32.33	-460.94 ± 28.76	-14.12 ± 1.98	410.88 ± 40.17	-475.06 ± 27.70	-64.18 ± 15.87

Table C. MM/PBSA binding energies (kcal/mol) for NOD1^{CARD}–NOD1^{CARD} and NOD2^{CARDa}–NOD2^{CARDa} homomeric ring interfaces

Interface | Trajectory		ΔE_{vdW}	ΔE_{ele}	ΔG_{solvp}	ΔG_{solvnp}	ΔE_{gas}	ΔG_{solv}	ΔG_{bind}
N1R2-F3 – NOD1^{CARD}-NOD1^{CARD} homomeric ring interface								
Homomer (Fig 2H)	T1	-206.30 ± 12.21	503.86 ± 25.10	-466.85 ± 20.37	-26.64 ± 1.16	297.56 ± 29.18	-493.49 ± 19.84	-195.93 ± 13.11
	T2	-220.97 ± 11.67	481.73 ± 20.80	-441.40 ± 18.71	-26.72 ± 1.07	260.76 ± 23.27	-468.11 ± 18.30	-207.35 ± 11.09
	T3	-199.60 ± 12.78	487.26 ± 25.62	-448.90 ± 22.83	-26.61 ± 1.23	287.67 ± 31.35	-475.51 ± 22.09	-187.84 ± 13.03
N2aR2-F3 – NOD2^{CARDa}-NOD2^{CARDa} homomeric ring interface								
Homomer (Fig 2H)	T1	-194.70 ± 22.99	978.29 ± 62.38	-907.96 ± 57.13	-22.20 ± 2.43	783.59 ± 72.72	-930.16 ± 55.90	-146.57 ± 25.46
	T2	-159.93 ± 17.40	1069.13 ± 54.33	-992.76 ± 50.49	-20.94 ± 1.74	909.20 ± 58.47	-1013.70 ± 50.10	-104.50 ± 16.09
	T3	-219.56 ± 18.78	934.03 ± 38.99	-856.42 ± 37.45	-27.31 ± 2.08	714.47 ± 49.80	-883.72 ± 36.43	-169.25 ± 18.36

Table D. MM/PBSA binding energies (kcal/mol) for NOD1–NOD1 homo-ring and NOD1–RIP2 ring–ring interfaces

Interface | Trajectory		ΔE_{vdW}	ΔE_{ele}	ΔG_{solvp}	ΔG_{solvnp}	ΔE_{gas}	ΔG_{solv}	ΔG_{bind}
N1C-N1C	T1	-252.89 ± 15.10	493.52 ± 22.60	-452.77 ± 17.88	-30.12 ± 1.09	240.63 ± 23.79	-482.90 ± 17.80	-242.27 ± 14.20
Homo-ring	T2	-265.88 ± 15.20	497.59 ± 26.99	-453.26 ± 22.80	-30.33 ± 1.33	231.71 ± 30.37	-483.60 ± 22.29	-251.89 ± 15.99
(Fig BD)	T3	-282.34 ± 14.83	511.65 ± 25.33	-457.54 ± 21.23	-32.30 ± 0.91	229.31 ± 25.41	-489.84 ± 21.11	-260.53 ± 12.82
N1C-R2C	T1	-338.19 ± 11.59	-149.46 ± 24.41	169.70 ± 19.54	-35.94 ± 0.80	-487.64 ± 25.58	133.76 ± 19.21	-353.88 ± 11.57
Top-down	T2	-313.98 ± 13.55	-138.53 ± 26.38	155.42 ± 24.13	-34.35 ± 1.03	-452.51 ± 32.95	121.07 ± 23.61	-331.44 ± 13.47
(Fig BE)	T3	-344.04 ± 11.96	-185.11 ± 20.60	207.12 ± 18.01	-37.73 ± 0.68	-529.14 ± 23.71	169.39 ± 17.77	-359.75 ± 11.26
N1C-R2C	T1	-234.41 ± 11.95	215.23 ± 22.62	-182.46 ± 19.42	-27.81 ± 1.04	-19.18 ± 25.11	-210.27 ± 19.04	-229.45 ± 10.91
Bottom-up	T2	-211.24 ± 12.53	222.33 ± 17.53	-190.23 ± 15.70	-24.50 ± 1.11	11.09 ± 22.90	-214.72 ± 15.18	-203.64 ± 12.41
(Fig BF)	T3	-286.60 ± 16.78	199.72 ± 21.12	-159.23 ± 18.31	-32.70 ± 1.31	-86.88 ± 27.48	-191.93 ± 17.69	-278.81 ± 15.43

Table E. MM/PBSA energy components (kcal/mol) of NOD1–NOD1 homodimeric and NOD1–RIP2 heterodimeric interfaces.

Interface | Trajectory		ΔE_{vdW}	ΔE_{ele}	ΔG_{solvp}	ΔG_{solvnp}	ΔE_{gas}	ΔG_{solv}	ΔG_{bind}
NOD1–NOD1 homodimeric interfaces								
Type-I (Fig CD)	T1	-35.16 ± 3.98	4.15 ± 6.56	-2.90 ± 5.34	-4.03 ± 0.22	-31.01 ± 6.83	-6.93 ± 5.31	-37.94 ± 3.37
	T2	-34.76 ± 3.83	3.49 ± 5.64	-1.69 ± 4.45	-4.12 ± 0.20	-31.27 ± 6.28	-5.80 ± 4.46	-37.07 ± 3.41
	T3	-22.81 ± 4.22	11.46 ± 9.23	-8.80 ± 7.49	-3.26 ± 0.32	-11.35 ± 10.05	-12.06 ± 7.37	-23.42 ± 4.23
Type-II (Fig CE)	T1	-29.16 ± 4.80	54.62 ± 7.19	-49.15 ± 6.02	-3.12 ± 0.41	25.46 ± 7.50	-52.27 ± 5.99	-26.80 ± 4.21
	T2	-30.29 ± 4.16	55.42 ± 5.69	-49.28 ± 5.04	-3.41 ± 0.30	25.12 ± 6.51	-52.69 ± 4.98	-27.56 ± 3.69
	T3	-44.07 ± 4.86	58.67 ± 9.11	-48.05 ± 7.31	-4.99 ± 0.41	14.59 ± 9.45	-53.04 ± 7.24	-38.44 ± 4.49
Type-III (Fig CF)	T1	-15.84 ± 2.66	16.04 ± 3.50	-16.06 ± 3.19	-2.06 ± 0.10	0.20 ± 4.05	-18.12 ± 3.19	-17.92 ± 2.22
	T2	-14.49 ± 2.27	19.21 ± 3.21	-19.69 ± 2.91	-2.02 ± 0.10	4.72 ± 3.60	-21.71 ± 2.90	-16.99 ± 1.87
	T3	-11.93 ± 2.89	-3.86 ± 5.10	-0.27 ± 3.88	-2.19 ± 0.12	-15.79 ± 5.04	-2.46 ± 3.87	-18.25 ± 2.42
$NOD1^{CARD}$– $RIP2^{CARD}$ heterodimeric top-down interfaces								
Type-I (Fig DD)	T1	-40.16 ± 4.46	-45.92 ± 10.51	45.69 ± 7.82	-4.87 ± 0.25	-86.09 ± 11.37	40.82 ± 7.67	-45.26 ± 5.06
	T2	-38.46 ± 3.99	-46.78 ± 8.49	44.82 ± 7.12	-4.53 ± 0.30	-85.24 ± 8.12	40.29 ± 7.01	-44.95 ± 3.21
	T3	-41.13 ± 3.85	-46.32 ± 8.74	45.78 ± 6.89	-4.59 ± 0.21	-87.46 ± 8.61	41.19 ± 6.82	-46.26 ± 3.58
Type-II (Fig DE)	T1	-53.36 ± 4.68	-29.65 ± 7.64	34.45 ± 6.73	-5.86 ± 0.34	-83.01 ± 8.96	28.59 ± 6.58	-54.42 ± 4.47
	T2	-41.43 ± 6.42	-21.47 ± 8.93	27.31 ± 8.79	-4.95 ± 0.47	-62.90 ± 12.27	22.36 ± 8.48	-40.54 ± 5.67
	T3	-44.34 ± 4.08	-20.19 ± 8.14	26.54 ± 6.94	-5.58 ± 0.31	-64.53 ± 8.59	20.96 ± 6.78	-43.57 ± 3.97
Type-III (Fig DF)	T1	-12.32 ± 3.51	-40.06 ± 5.41	34.68 ± 4.16	-2.61 ± 0.13	-52.38 ± 5.30	32.06 ± 4.14	-20.31 ± 2.77
	T2	-10.26 ± 3.08	-47.36 ± 4.30	39.70 ± 3.64	-2.34 ± 0.13	-57.61 ± 4.87	37.35 ± 3.63	-20.26 ± 2.62
	T3	-10.79 ± 3.30	-41.63 ± 6.01	34.83 ± 4.47	-2.21 ± 0.13	-52.42 ± 6.22	32.62 ± 4.44	-19.79 ± 2.89
$NOD1^{CARD}$–$RIP2^{CARD}$ heterodimeric bottom-top interfaces								
Type-I (Fig ED)	T1	-15.61 ± 5.46	28.15 ± 6.34	-24.75 ± 5.01	-2.19 ± 0.40	12.54 ± 6.48	-26.94 ± 5.05	-14.40 ± 4.46
	T2	-32.49 ± 4.05	7.36 ± 7.51	-4.23 ± 6.31	-3.61 ± 0.29	-25.12 ± 8.21	-7.85 ± 6.27	-32.97 ± 3.73
	T3	-33.36 ± 4.21	12.67 ± 8.06	-7.39 ± 6.81	-3.85 ± 0.30	-20.68 ± 9.37	-11.24 ± 6.71	-31.92 ± 4.29
Type-II (Fig EE)	T1	-27.92 ± 6.64	19.97 ± 10.06	-14.94 ± 9.88	-3.57 ± 0.76	-7.95 ± 13.07	-18.51 ± 9.42	-26.46 ± 6.09
	T2	-31.30 ± 5.22	9.39 ± 10.35	-4.92 ± 8.40	-4.03 ± 0.56	-21.90 ± 10.89	-8.95 ± 8.43	-30.86 ± 4.59
	T3	-43.63 ± 4.78	27.60 ± 10.48	-19.55 ± 8.93	-5.30 ± 0.55	-16.03 ± 12.60	-24.85 ± 8.51	-40.88 ± 5.50
Type-III (Fig EF)	T1	-16.54 ± 2.57	-10.23 ± 4.60	9.82 ± 4.44	-2.42 ± 0.23	-26.78 ± 5.51	7.41 ± 4.33	-19.37 ± 2.15
	T2	-16.07 ± 3.13	-11.20 ± 7.86	10.06 ± 6.22	-2.38 ± 0.18	-27.27 ± 7.65	7.68 ± 6.17	-19.58 ± 2.83
	T3	-22.04 ± 3.25	-14.69 ± 8.14	13.98 ± 6.44	-2.96 ± 0.22	-36.73 ± 8.00	11.01 ± 6.38	-25.71 ± 2.87

Table F. MM/PBSA binding energies (kcal/mol) for NOD2^{CARDa}–NOD2^{CARDb}, NOD2^{CARDa}–RIP2^{CARD,} and NOD2^{CARDb}–RIP2^{CARD} ring interfaces

Interface | Trajectory		ΔE_{vdW}	ΔE_{ele}	ΔG_{solvp}	ΔG_{solvnp}	ΔE_{gas}	ΔG_{solv}	ΔG_{bind}
N2Ca-N2Cb	T1	-382.82 ± 20.11	880.26 ± 33.29	-792.97 ± 30.79	-42.79 ± 1.79	497.44 ± 42.60	-835.76 ± 29.88	-338.32 ± 19.16
Hetero-ring	T2	-373.19 ± 32.30	937.03 ± 39.03	-836.59 ± 35.92	-41.97 ± 3.46	563.84 ± 61.60	-878.55 ± 33.95	-314.71 ± 34.71
(Fig ID)	T3	-343.12 ± 17.82	905.38 ± 36.90	-815.23 ± 31.47	-39.20 ± 1.65	562.27 ± 40.82	-854.43 ± 30.85	-292.16 ± 17.10
N2Ca-R2C	T1	-285.64 ± 24.01	-37.08 ± 30.47	62.75 ± 29.40	-33.61 ± 2.18	-322.72 ± 45.62	29.14 ± 27.83	-293.58 ± 22.40
Top-down	T2	-320.24 ± 14.38	-96.85 ± 21.86	113.07 ± 18.29	-36.40 ± 0.98	-417.09 ± 26.70	76.67 ± 17.98	-340.42 ± 14.60
(Fig IE)	T3	-245.79 ± 18.43	-27.97 ± 24.88	48.70 ± 22.96	-30.37 ± 1.33	-273.76 ± 33.99	18.33 ± 22.43	-255.44 ± 17.62
N2Cb-R2C	T1	-256.91 ± 16.98	408.13 ± 32.64	-353.80 ± 27.94	-30.35 ± 1.88	151.23 ± 39.55	-384.15 ± 26.99	-232.93 ± 18.11
Bottom-up	T2	-312.34 ± 17.87	319.58 ± 22.84	-263.23 ± 20.85	-34.56 ± 1.62	7.24 ± 31.51	-297.79 ± 20.18	-290.55 ± 17.25
(Fig IF)	T3	-325.29 ± 13.75	362.74 ± 23.23	-304.22 ± 20.74	-36.12 ± 1.16	37.45 ± 27.25	-340.34 ± 20.33	-302.90 ± 12.80

Table G. MM/PBSA binding energies (kcal/mol) of homo– and hetero–CARD interfaces within NOD2 tandem CARD rings.

Interface | Trajectory		ΔE_{vdW}	ΔE_{ele}	ΔG_{solvp}	ΔG_{solvnp}	ΔE_{gas}	ΔG_{solv}	ΔG_{bind}
NOD2^{CARDa}–NOD2^{CARDb} tandem and heterodimeric interfaces (within the ring)								
Tandem	T1	-57.28 ± 5.04	20.24 ± 6.47	-9.11 ± 5.48	-5.57 ± 0.48	-37.04 ± 7.60	-14.68 ± 5.23	-51.72 ± 4.55
Type-II	T2	-38.02 ± 5.41	-12.48 ± 9.54	16.26 ± 7.51	-5.12 ± 0.42	-50.50 ± 9.62	11.14 ± 7.53	-39.35 ± 4.60
(Fig JD)	T3	-33.76 ± 9.82	12.39 ± 7.19	-7.03 ± 6.82	-3.87 ± 1.10	-21.37 ± 15.51	-10.91 ± 6.03	-32.28 ± 10.87
Type-I	T1	-51.62 ± 4.45	-112.20 ± 8.84	102.00 ± 7.52	-5.38 ± 0.35	-163.83 ± 9.93	96.62 ± 7.41	-67.20 ± 4.52
(Fig JE)	T2	-63.28 ± 7.07	-86.07 ± 14.74	84.60 ± 10.80	-6.70 ± 0.60	-149.35 ± 11.16	77.90 ± 11.05	-71.45 ± 4.52
	T3	-43.50 ± 8.73	-124.78 ± 20.55	117.18 ± 16.21	-5.10 ± 0.61	-168.28 ± 22.70	112.08 ± 15.88	-56.19 ± 10.02
Type-III	T1	-7.64 ± 3.11	-1.06 ± 3.58	1.97 ± 3.83	-0.99 ± 0.46	-8.70 ± 6.14	0.98 ± 3.50	-7.72 ± 3.06
(Fig JF)	T2	-20.45 ± 4.31	3.89 ± 3.63	N/A	-2.25 ± 0.33	-16.56 ± 4.59	-2.24 ± 2.90	-18.81 ± 4.00
	T3	-5.56 ± 2.58	4.92 ± 3.46	-3.98 ± 3.01	-0.65 ± 0.42	-0.65 ± 3.18	-4.63 ± 3.19	-5.27 ± 2.38
NOD2^{CARDa}–NOD2^{CARDa} homodimeric interfaces (within the ring)								
Type-I	T1	-57.63 ± 3.87	21.26 ± 8.75	-10.98 ± 7.28	-5.80 ± 0.33	-36.37 ± 9.25	-16.79 ± 7.18	-53.16 ± 4.07
(Fig KE)	T2	-44.32 ± 4.94	12.01 ± 13.60	-6.84 ± 10.16	-5.24 ± 0.22	-32.30 ± 11.57	-12.08 ± 10.17	-44.38 ± 3.34
	T3	-26.09 ± 5.27	-1.58 ± 10.72	-1.83 ± 8.67	-3.99 ± 0.52	-27.66 ± 12.37	-5.82 ± 8.49	-33.48 ± 5.61
Type-III	T1	-8.40 ± 2.97	54.74 ± 4.44	-51.89 ± 3.76	-1.24 ± 0.24	46.34 ± 4.46	-53.13 ± 3.77	-6.79 ± 2.65
(Fig KF)	T2	-5.05 ± 1.84	67.24 ± 5.41	-62.84 ± 4.80	-0.62 ± 0.31	62.18 ± 5.02	-63.46 ± 4.85	-1.28 ± 1.57
	T3	-5.61 ± 2.06	59.10 ± 5.28	-55.73 ± 4.83	-0.82 ± 0.26	53.49 ± 5.49	-56.55 ± 4.81	-3.06 ± 2.08
NOD2^{CARDb}–NOD2^{CARDb} homodimeric interfaces (within the ring)								
Type-I	T1	-50.80 ± 3.97	-57.72 ± 5.67	56.36 ± 4.95	-4.90 ± 0.37	-108.52 ± 7.02	51.46 ± 4.79	-57.07 ± 3.68
(Fig KG)	T2	-62.47 ± 6.27	-47.87 ± 7.74	50.76 ± 6.96	-5.91 ± 0.57	-110.34 ± 10.41	44.85 ± 6.65	-65.49 ± 5.83
	T3	-39.82 ± 10.10	-46.28 ± 10.57	47.71 ± 9.93	-4.79 ± 0.83	-86.10 ± 16.99	42.92 ± 9.38	-43.17 ± 9.14
Type-III	T1	-19.16 ± 3.92	-20.82 ± 4.16	21.14 ± 3.33	-2.49 ± 0.43	-39.97 ± 4.88	18.65 ± 3.43	-21.33 ± 3.77
(Fig KH)	T2	-15.67 ± 2.85	-12.97 ± 4.96	14.51 ± 4.60	-1.99 ± 0.29	-28.64 ± 6.57	12.52 ± 4.44	-16.12 ± 3.10
	T3	-10.07 ± 4.01	-33.64 ± 7.84	30.29 ± 6.37	-1.75 ± 0.43	-43.71 ± 8.20	28.54 ± 6.23	-15.17 ± 3.72

Table H. MM/PBSA binding energies (kcal/mol) of NOD2–RIP2 hetero–CARD dimeric interfaces

Interface | Trajectory		ΔE_{vdW}	ΔE_{ele}	ΔG_{solvp}	ΔG_{solvnp}	ΔE_{gas}	ΔG_{solv}	ΔG_{bind}
$NOD2^{CARDa}$–$NOD2^{CARDb}$ heterodimeric interfaces								
Type-I (Fig LD)	T1	-44.64 ± 8.06	-7.57 ± 5.27	14.08 ± 5.04	-5.38 ± 0.54	-52.21 ± 11.22	8.69 ± 4.69	-43.51 ± 7.75
	T2	-54.87 ± 5.98	-34.91 ± 7.23	38.64 ± 5.38	-6.27 ± 0.35	-89.78 ± 7.61	32.37 ± 5.43	-57.42 ± 5.53
	T3	-35.00 ± 7.55	-11.50 ± 9.07	17.01 ± 7.48	-4.34 ± 0.50	-46.50 ± 12.38	12.67 ± 7.28	-33.83 ± 7.14
Type-II (Fig LE)	T1	-66.74 ± 8.40	27.37 ± 9.80	-14.53 ± 7.84	-7.35 ± 0.86	-39.38 ± 12.53	-21.89 ± 7.50	-61.26 ± 8.19
	T2	-43.23 ± 5.81	8.62 ± 10.18	-1.14 ± 9.05	-5.11 ± 0.57	-34.61 ± 12.52	-6.24 ± 8.72	-40.85 ± 5.66
	T3	-41.62 ± 6.97	10.71 ± 14.23	-3.36 ± 12.35	-5.38 ± 0.74	-30.91 ± 13.85	-8.74 ± 12.45	-39.65 ± 6.29
Type-III (Fig LF)	T1	-12.27 ± 3.65	-68.99 ± 6.39	61.83 ± 5.30	-2.41 ± 0.21	-81.26 ± 7.45	59.42 ± 5.21	-21.83 ± 3.61
	T2	-22.07 ± 3.84	-60.33 ± 10.27	59.34 ± 8.44	-3.14 ± 0.38	-82.40 ± 10.50	56.20 ± 8.35	-26.19 ± 3.81
	T3	-12.36 ± 3.67	-65.83 ± 9.79	60.26 ± 8.94	-2.32 ± 0.26	-78.19 ± 10.21	57.93 ± 8.80	-20.25 ± 3.16
$NOD2^{CARDa}$–$RIP2^{CARD}$ heterodimeric top-down interfaces								
Type-I (Fig MD)	T1	-30.00 ± 6.37	-4.34 ± 5.64	8.82 ± 4.66	-3.67 ± 0.42	-34.34 ± 8.30	5.15 ± 4.54	-29.19 ± 5.67
	T2	-25.02 ± 8.15	-7.08 ± 6.95	9.29 ± 5.81	-3.47 ± 0.53	-32.10 ± 9.02	5.82 ± 5.73	-26.28 ± 7.18
	T3	-31.92 ± 5.28	-10.52 ± 6.47	14.05 ± 6.82	-4.47 ± 0.43	-42.44 ± 9.26	9.57 ± 6.59	-32.87 ± 4.11
Type-II (Fig ME)	T1	-49.89 ± 6.98	-15.31 ± 7.49	24.11 ± 6.97	-5.59 ± 0.51	-65.20 ± 10.79	18.52 ± 6.73	-46.68 ± 6.10
	T2	-51.91 ± 4.34	-17.95 ± 8.34	23.60 ± 6.99	-5.39 ± 0.40	-69.87 ± 9.11	18.21 ± 6.84	-51.66 ± 4.45
	T3	-52.62 ± 6.57	-25.82 ± 9.92	33.31 ± 8.76	-5.78 ± 0.53	-78.44 ± 10.03	27.53 ± 8.58	-50.91 ± 6.00
Type-III (Fig MF)	T1	-12.98 ± 3.54	-43.53 ± 5.24	38.16 ± 4.25	-2.37 ± 0.26	-56.51 ± 5.63	35.79 ± 4.25	-20.72 ± 2.98
	T2	-17.83 ± 3.82	-64.82 ± 6.86	56.52 ± 5.89	-3.44 ± 0.22	-82.64 ± 7.32	53.07 ± 5.88	-29.57 ± 3.45
	T3	-9.11 ± 3.58	-39.63 ± 13.33	35.20 ± 11.43	-2.15 ± 0.29	-48.74 ± 13.69	33.05 ± 11.26	-15.69 ± 3.46
$NOD2^{CARDb}$–$RIP2^{CARD}$ heterodimeric bottom-top interfaces								
Type-I (Fig ND)	T1	-44.29 ± 4.33	-38.67 ± 6.04	37.38 ± 4.95	-5.44 ± 0.25	-82.96 ± 6.20	31.94 ± 4.97	-51.02 ± 3.49
	T2	-47.30 ± 4.23	-33.94 ± 7.29	39.33 ± 5.74	-5.20 ± 0.20	-81.24 ± 7.65	34.13 ± 5.66	-47.11 ± 4.12
	T3	-43.74 ± 4.99	-43.40 ± 7.97	42.16 ± 6.31	-5.01 ± 0.31	-87.14 ± 8.77	37.15 ± 6.22	-50.00 ± 4.69
Type-II (Fig NE)	T1	-35.83 ± 5.59	16.63 ± 8.90	-7.91 ± 8.45	-4.52 ± 0.65	-19.20 ± 10.97	-12.43 ± 8.24	-31.63 ± 5.06
	T2	-31.22 ± 8.21	-9.65 ± 9.12	14.40 ± 7.99	-4.45 ± 0.94	-40.87 ± 11.43	9.95 ± 7.91	-30.92 ± 7.64
	T3	-16.55 ± 3.30	3.67 ± 5.86	-0.42 ± 5.37	-1.96 ± 0.41	-12.87 ± 6.45	-2.38 ± 5.37	-15.25 ± 3.19
Type-III (Fig NF)	T1	-16.56 ± 3.30	-55.42 ± 5.14	51.93 ± 4.90	-2.30 ± 0.17	-71.98 ± 6.73	49.63 ± 4.85	-22.36 ± 2.93
	T2	-17.09 ± 2.73	-45.93 ± 6.26	42.59 ± 5.16	-2.28 ± 0.20	-63.01 ± 6.69	40.31 ± 5.12	-22.71 ± 2.68
	T3	-19.55 ± 3.12	-29.62 ± 8.12	29.85 ± 6.80	-2.58 ± 0.29	-49.17 ± 7.99	27.27 ± 6.67	-21.90 ± 2.71

Table I. List Residues mutated at different interface types in NOD1–RIP2 and NOD2–RIP2 top-down and bottom-up complexes.

NOD1–RIP2^{mTD} (Fig 7A)			NOD1–RIP2^{mBU} (Fig 7B)		
Subunit	Interface	Mutations	Subunit	Interface	Mutations
NOD1^{CARD}	Type-Ia	Q21E, K24E, R27E, R69E	NOD1^{CARD}	Type-Ib	N43D, K46E
	Type-IIa	K70E, S77A, K78E		Type-IIb	R35E, N36D
	Type-IIIa	–NA–		Type-IIIb	Q64E, R69E
RIP2^{CARD}	Type-Ib	Y474A, K508E	RIP2^{CARD}	Type-Ia	R444E, R488E
	Type-IIb	Q458A, R483E		Type-IIa	–NA–
	Type-IIIb	R488E		Type-IIIa	–NA–
NOD2–RIP2^{mTD} (Fig 7C)			NOD2–RIP2^{mBU} (Fig 7D)		
NOD2^{CARDa}	Type-Ia	R38E,R86E	NOD2^{CARDb}	Type-Ib	E158A, R171E
	Type-IIa	E69A, R87A, N94A, K95A, W98A		Type-IIb	R143E, R144A
	Type-IIIa	–NA–		Type-IIIb	R182E, T176A
RIP2^{CARD}	Type-Ib	Y474A, K471E	RIP2^{CARD}	Type-Ia	R444E, R488E
	Type-IIb	Q458A, R483E, K513A, M515A		Type-IIa	–NA–
	Type-IIIb	R444E, R488E, T484A		Type-IIIa	–NA–
