# Supplementary material for: Structural and dynamic basis of NOD2 tandem CARD association and NOD1/2–RIP2 signaling complexes
Source: PLoS Comput Biol. 2026 May 29;22(5):e1014311. doi: 10.1371/journal.pcbi.1014311 (PMC13249360; doi:10.1371/journal.pcbi.1014311)
Supplement: S2 File — (PDF) [file pcbi.1014311.s002.pdf]

Supporting Information | S2 File (Supporting Figures)

Structural and Dynamic Basis of NOD2 Tandem CARD Association and NOD1/2–RIP2 Signaling Complexes

Jitendra Maharana[1,2,3,*], Aritra Bej[4,5], Debasish Biswal[6,7,8], Debashis Panda[9], and Arjun Sharma[10,*]

[1]Institute of Biological Chemistry, Academia Sinica, Taipei 11529, Taiwan. [2]Taiwan International Graduate Program (TIGP), Chemical Biology and Molecular Biophysics (CBMB), Academia Sinica, Taipei, 11529, Taiwan. [3]Institute of Bioinformatics and Structural Biology, College of Life Sciences and Medicine, National Tsing Hua University, Hsinchu 300, Taiwan. [4]Department of Chemistry and [5]Department of Pharmacology, University of California, Davis, CA, 95616, USA. [6]Institute of Molecular Biology, Academia Sinica, Taipei 11529, Taiwan. [7]Taiwan International Graduate Program (TIGP) - Interdisciplinary Neuroscience (INS), Academia Sinica, 11529, Taiwan. [8]College of Life Science, National Taiwan University, Taipei, 10617, Taiwan. [9]DBT-APSCS&T, Centre of Excellence for Bioresources and Sustainable Development, Kimin, Arunachal Pradesh 791121, India. [10]Department of Chemistry and Biochemistry, Purdue University Fort Wayne, Fort Wayne, Indiana 46805, USA.

*Correspondence: jitued@gmail.com (J.M.); arjun.sharma@pfw.edu (A.S.)

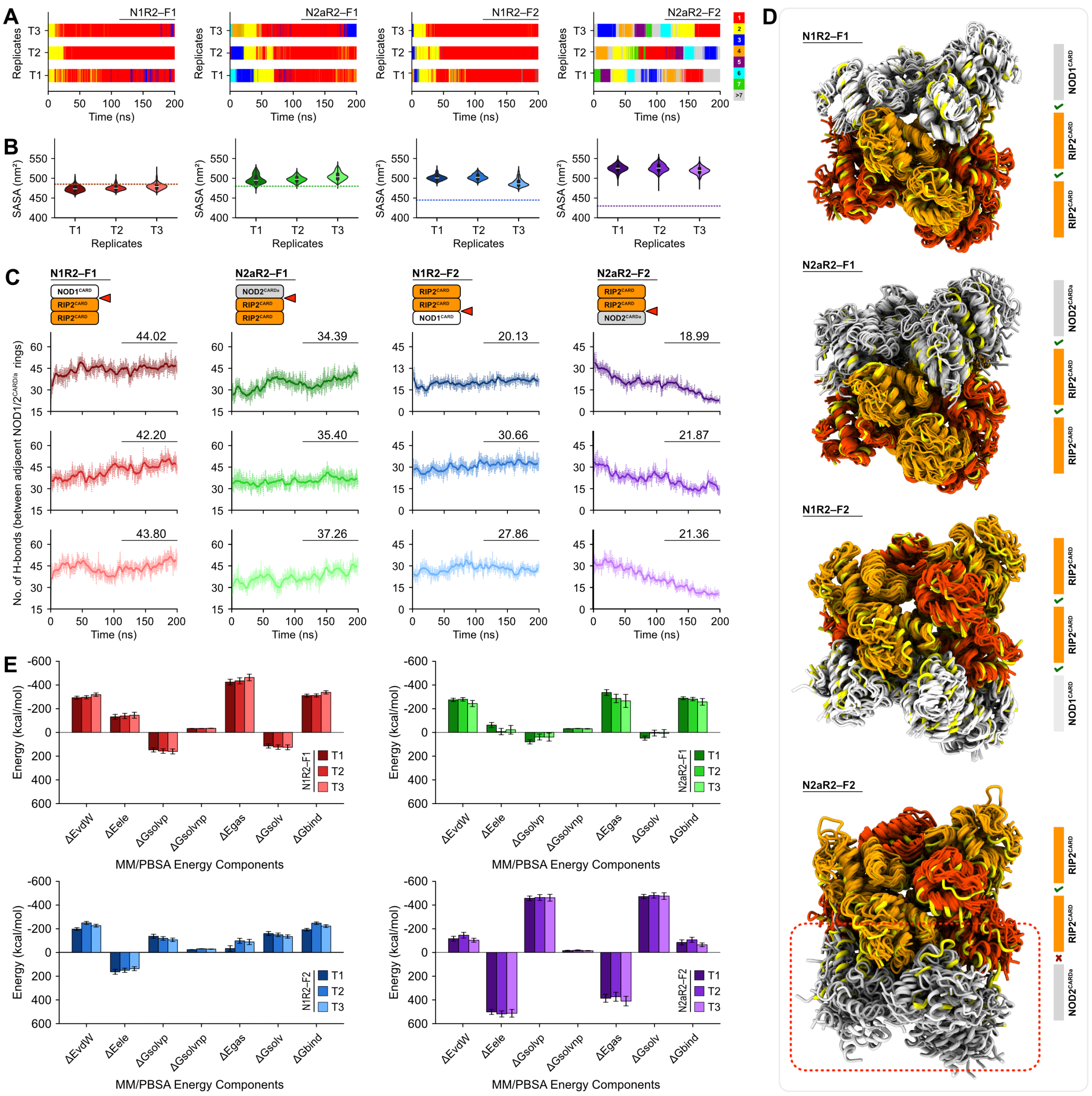

Fig. A. MD analysis of NOD1/2–RIP2 hetero-CARD filament models. (A) RMSD-based clustering over time for N1R2–F1, N2aR2–F1, N1R2–F2, and N2aR2–F2 across three independent trajectories (T1–T3); each color represents a distinct conformational cluster, with red indicating the dominant state. **(B)** Violin plots of solvent-accessible surface area (SASA) for each filament model, showing distributions across T1–T3. **(C)** Time evolution of intermolecular hydrogen bonds between NOD1/2 and RIP2 rings for each filament model, with mean H-bond counts indicated. **(D)** Structural ensembles of the four filament models; pre-MD structures are shown in yellow and superposed MD snapshots in color, illustrating conformational stability or drift. Favorable ring–ring interfaces are marked with green checkmarks, and incompatible interfaces with red crosses. **(E)** MM/PBSA binding free energy and its components for the ring–ring interfaces in each filament model across the three trajectories (see Table B).

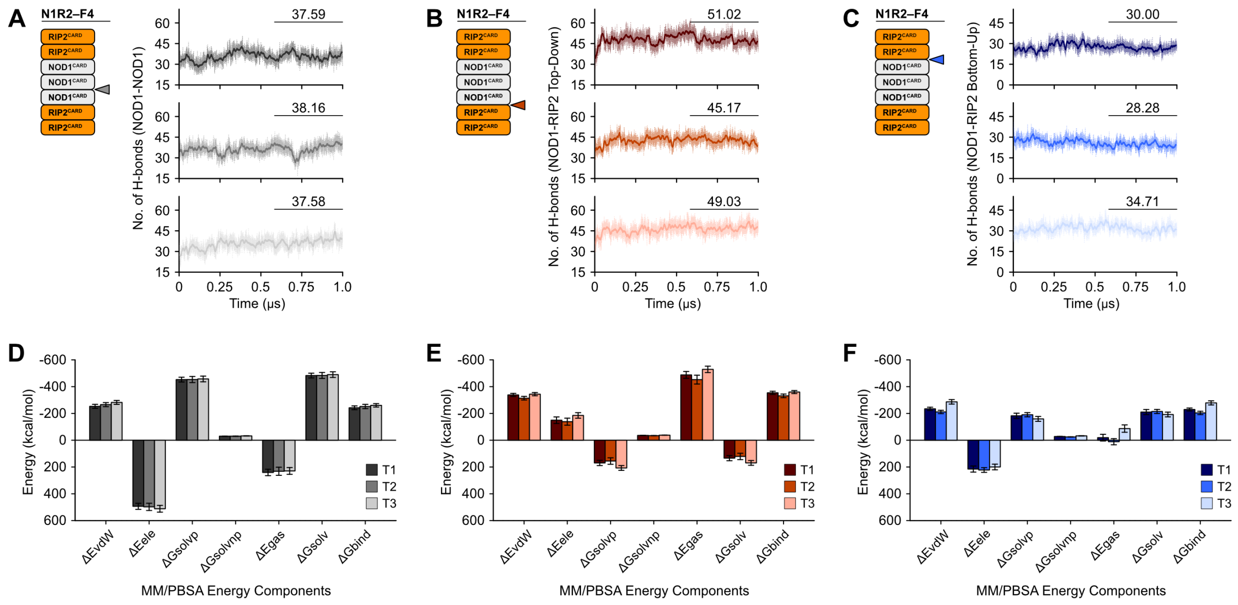

Fig B. Time-dependent H-bonds and energetics at NOD1–NOD1 and NOD1–RIP2 ring interfaces. (A–C) Number of intermolecular H-bonds over time for the NOD1–NOD1 interface **(A)**, NOD1–RIP2 top-down interface **(B)**, and NOD1–RIP2 bottom-up interface **(C)** over three independent 1 µs simulations, with average H-bonds shown. (D–F) MM/PBSA binding free-energy components and total binding free energies (ΔG_{bind}) for the same interfaces (A–C), shown for each trajectory (T1–T3), as summarized (in Table D).

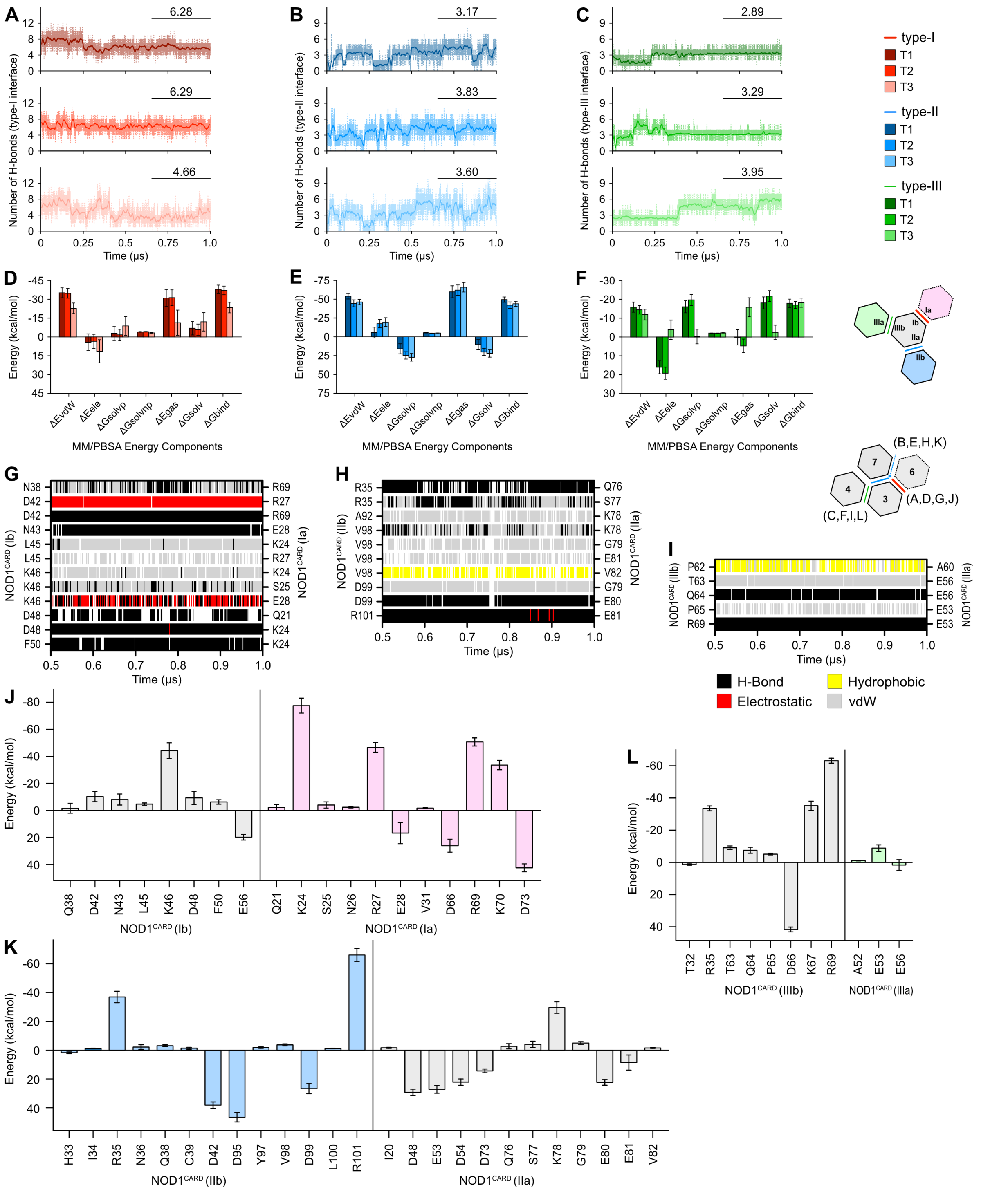

Fig C. Stability and energetics at NOD1^{CARD}–NOD1^{CARD} homodimeric interfaces. (A–C) Time evolution of intermolecular H-bonds at type-I **(A)**, type-II **(B)**, and type-III **(C)** interfaces over three 1 μs trajectories. **(D–F)** MM/PBSA binding free-energy components for type-I **(D)**, type-II **(E)**, and type-III **(F)** interfaces (see Tabe D). **(G–I)** Residue–residue contact maps for representative type-I **(G)**, type-II **(H)**, and type-III **(I)** homodimers, colored by contact type: H-bonds (black), electrostatic contacts (red), hydrophobic contacts (yellow), and van der Waals contacts (gray). **(J–L)** Per-residue MM/PBSA energy decomposition at type-I **(J)**, type-II **(K)**, and type-III **(L)** interfaces (Table E), highlighting energetic hot-spot residues.

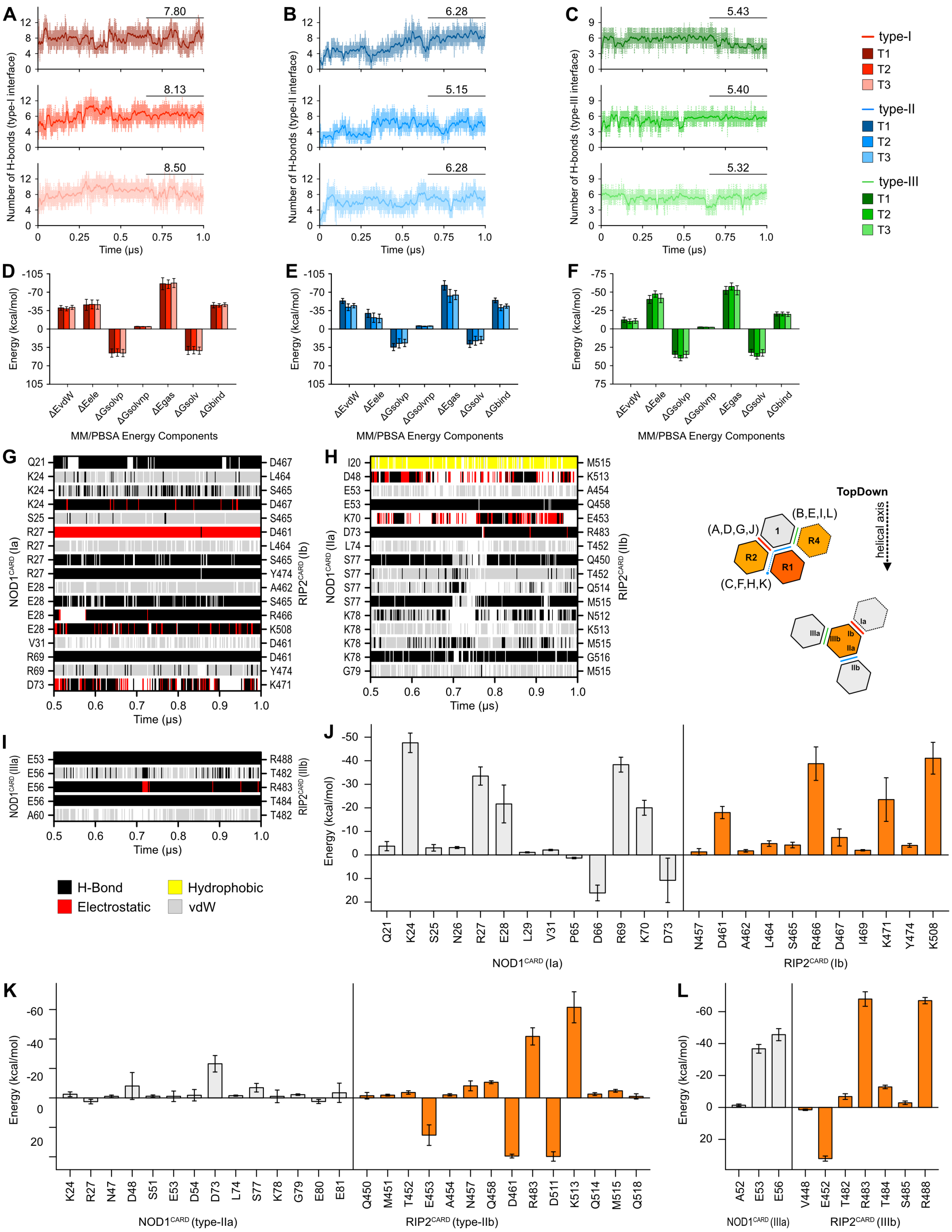

Fig D. Stability and energetics at NOD1^{CARD}–RIP2^{CARD} top-down heterodimeric interfaces. (A–C) Time evolution of intermolecular H-bonds for type-I **(A)**, type-II **(B)**, and type-III **(C)** NOD1-RIP2 interfaces over three 1 μs trajectories. **(D–F)** MM/PBSA binding free-energy components for type-I **(D)**, type-II **(E)**, and type-III **(F)** interfaces (Table D). **(G–I)** Residue–residue contact maps for representative type-I **(G)**, type-II **(H)**, and type-III **(I)** heterodimers, with H-bonds (black), electrostatic contacts (red), hydrophobic contacts (yellow), and vdW contacts (gray). **(J–L)** Per-residue MM/PBSA energy decomposition for monomers at type-I **(J)**, type-II **(K)**, and type-III **(L)** interfaces (Table E), identifying key energetic contributors.

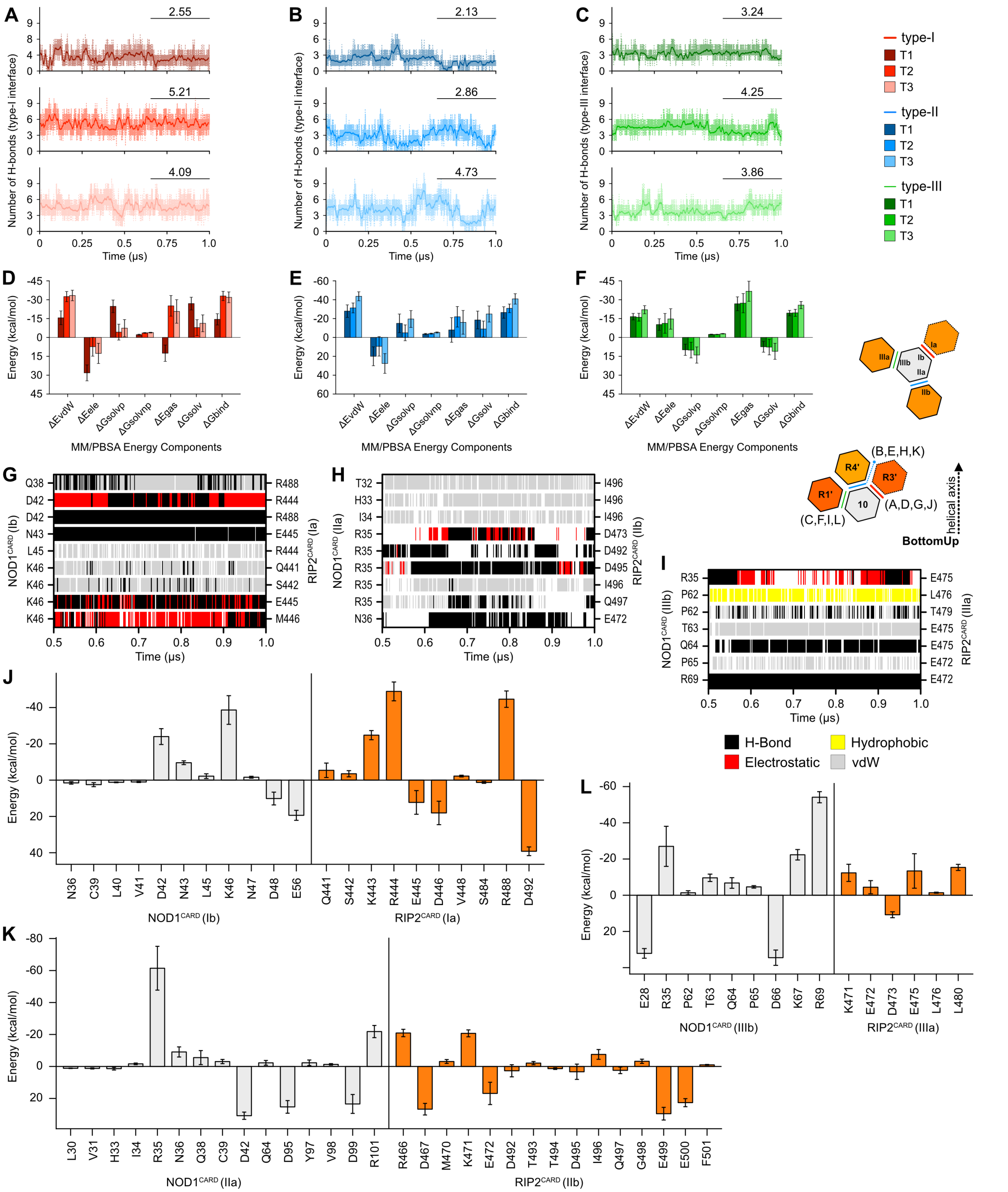

Fig E. Stability and energetics at NOD1^{CARD}–RIP2^{CARD} bottom-up heterodimeric interfaces. (A–C) Time evolution of intermolecular H-bond numbers for type-I **(A)**, type-II **(B)**, and type-III **(C)** interfaces across three 1 μs trajectories. **(D–F)** MM/PBSA binding free-energy components for type-I (D), type-II (E), and type-III **(F)** interfaces (see Table D). **(G–I)** Residue–residue contact maps for representative type-I **(G)**, type-II **(H)**, and type-III **(I)** heterodimers, with contact types with H-bonds (black), electrostatic contacts (red), hydrophobic contacts (yellow), and vdW contacts (gray). **(J–L)** Per-residue MM/PBSA energy decomposition for monomer at type-I **(J)**, type-II **(K)**, and type-III **(L)** interfaces (Table E), highlighting residues that dominate binding.

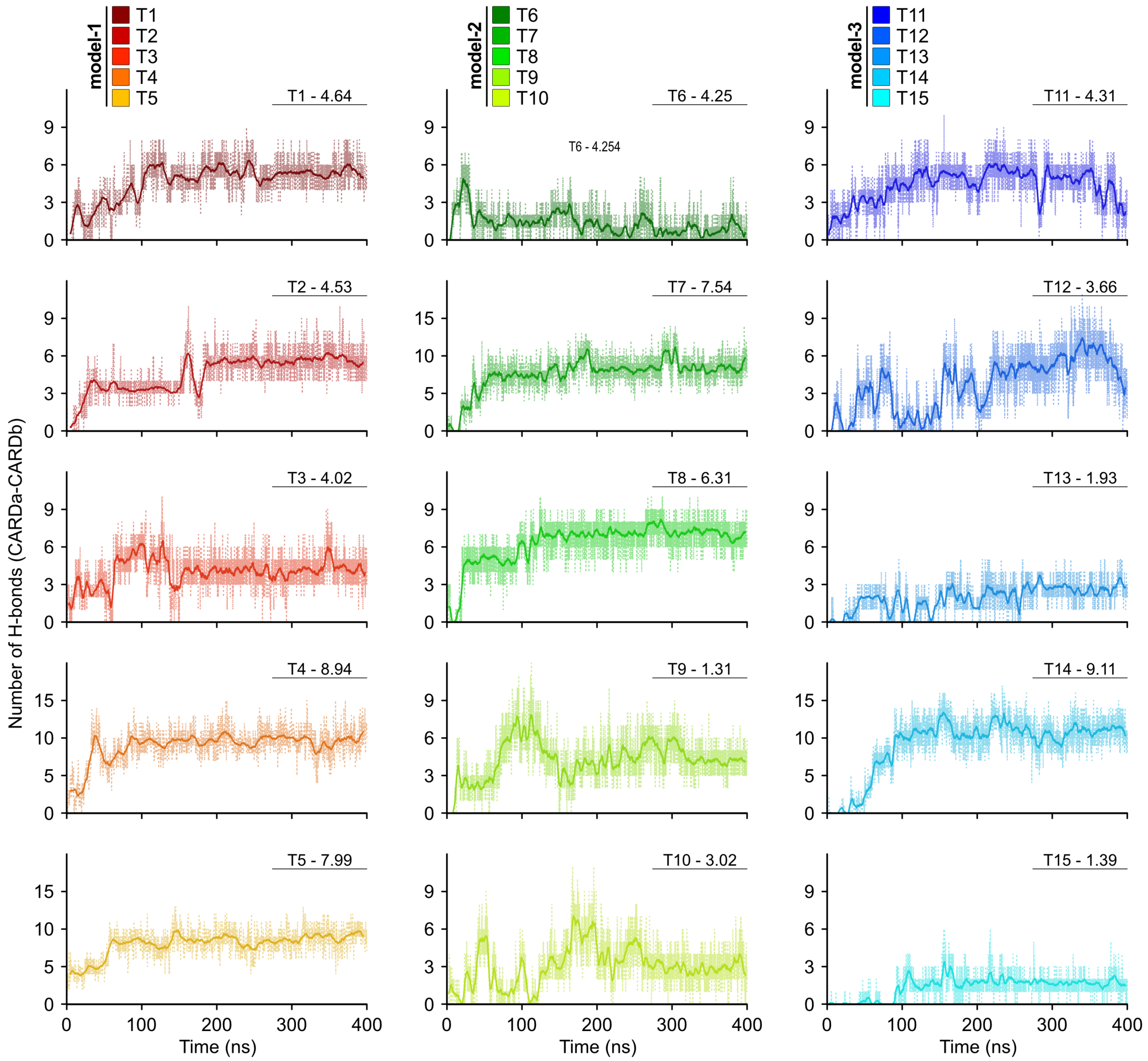

Fig F. H-bond dynamics at the NOD2 tandem CARD interface. Change in the total number of H-bonds between CARDa and CARDb at the tandem interface over simulation time. Plots show H-bond counts for model-1, model-2, and model-3 NOD2 tandem CARD constructs, colored in red, green, and blue, respectively, with total bond counts indicated, illustrating differences in interdomain association across models and trajectories.

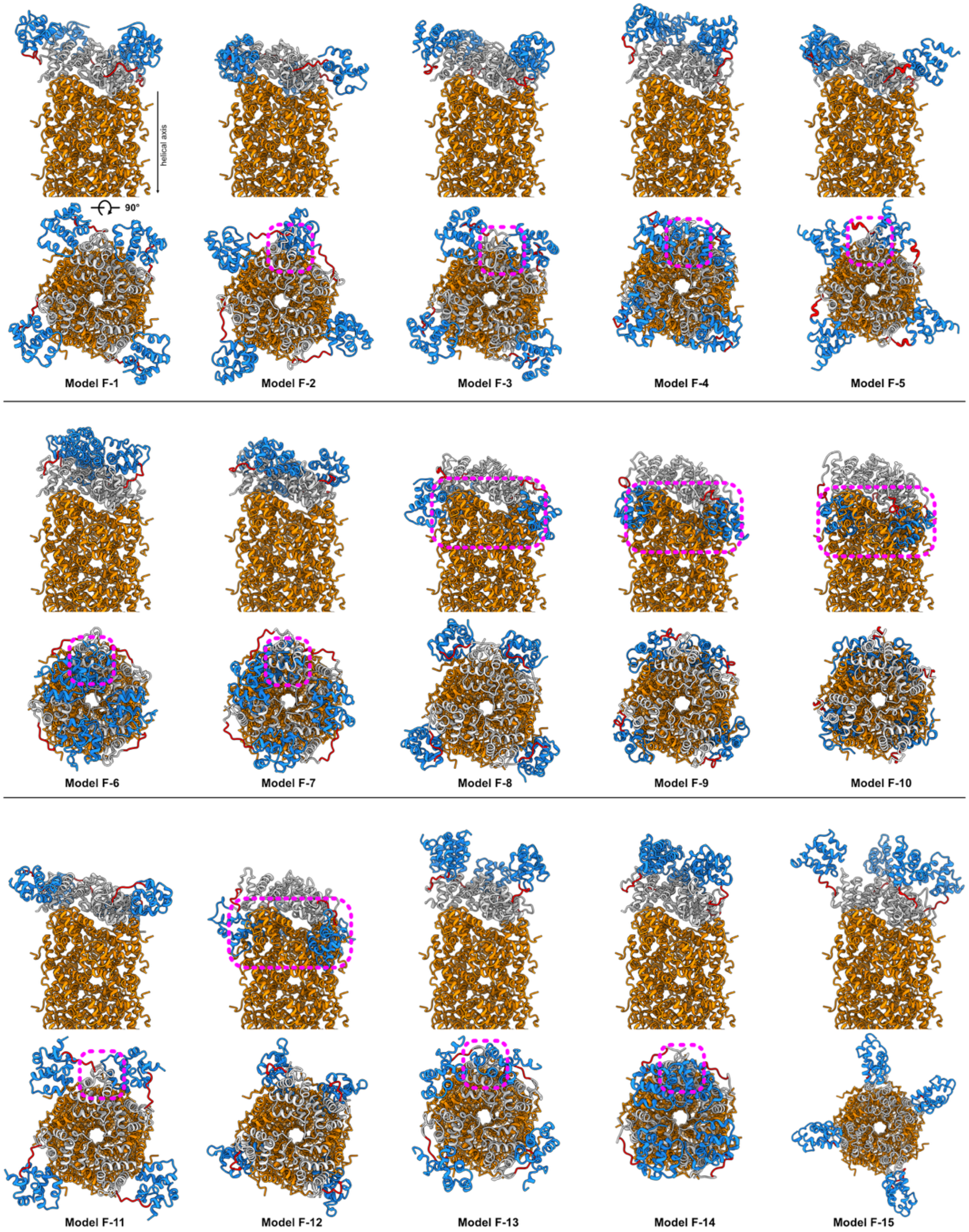

Fig G. Geometric compatibility of NOD2^{CARDab}–RIP2^{CARD} filament models. Side and top views of 15 NOD2^{CARDab}–RIP2^{CARD} filament model constructs generated by placing NOD2 tandem CARD conformations onto the RIP2^{CARD} filament. The panels illustrate how different NOD2^{CARDab} conformations position CARDa and CARDb relative to RIP2^{CARD} rings. All steric clashes, either between two NOD2^{CARDab} subunits or between NOD2^{CARDab} and RIP2^{CARD}, are highlighted with magenta boxes.

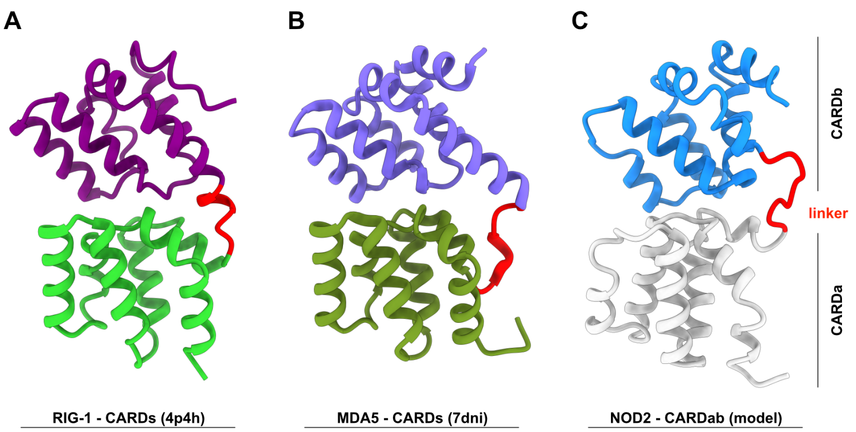

Fig H. Modeling NOD2 tandem CARDs from RIG-1 and MDA5 templates. Comparative structural overview of **(A)** RIG-I (PDB ID: 4P4H), **(B)** MDA5 (PDB ID: 7DNI), and **(C)** NOD2 tandem CARDs models. In each case, the CARD domains are shown as differently colored cartoons, and the inter-CARD linker is shown in red. The figure highlights similarities and differences in domain and linker organization.

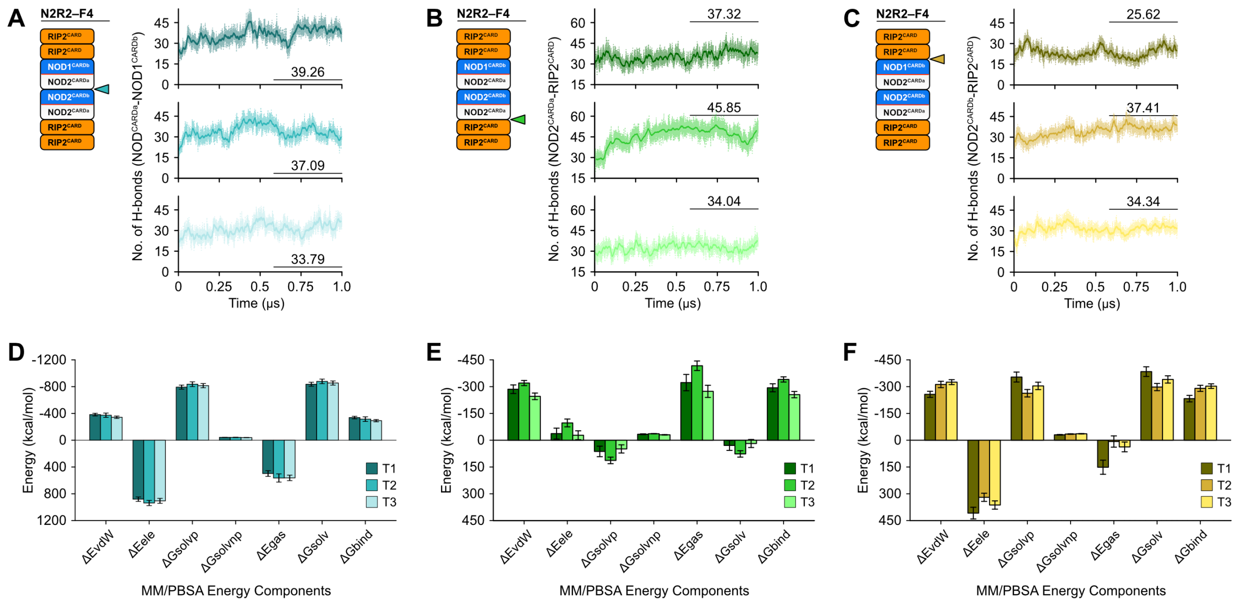

Fig I. Stability of NOD2^{CARDab}–RIP2^{CARD} ring–ring interfaces in N2R2–F4 filament model. (A–C) Time-dependent H-bond counts for the NOD2^{CARDa}–NOD2^{CARDb} hetero–ring interface **(A)**, the top-down NOD2^{CARDa}-RIP2^{CARD} interface **(B)**, and the bottom-up NOD2^{CARDb}–RIP2^{CARD} interface **(C)**, respectively. **(D–F)** MM/PBSA binding free energies and component contributions for these three interfaces, as summarized (in Table F); **(D)** NOD2^{CARDa}–NOD2^{CARDb}, **(E)** top-down NOD2^{CARDa}–RIP2^{CARD}, and **(F)** NOD2^{CARDb}–RIP2^{CARD} bottom-up interfaces.

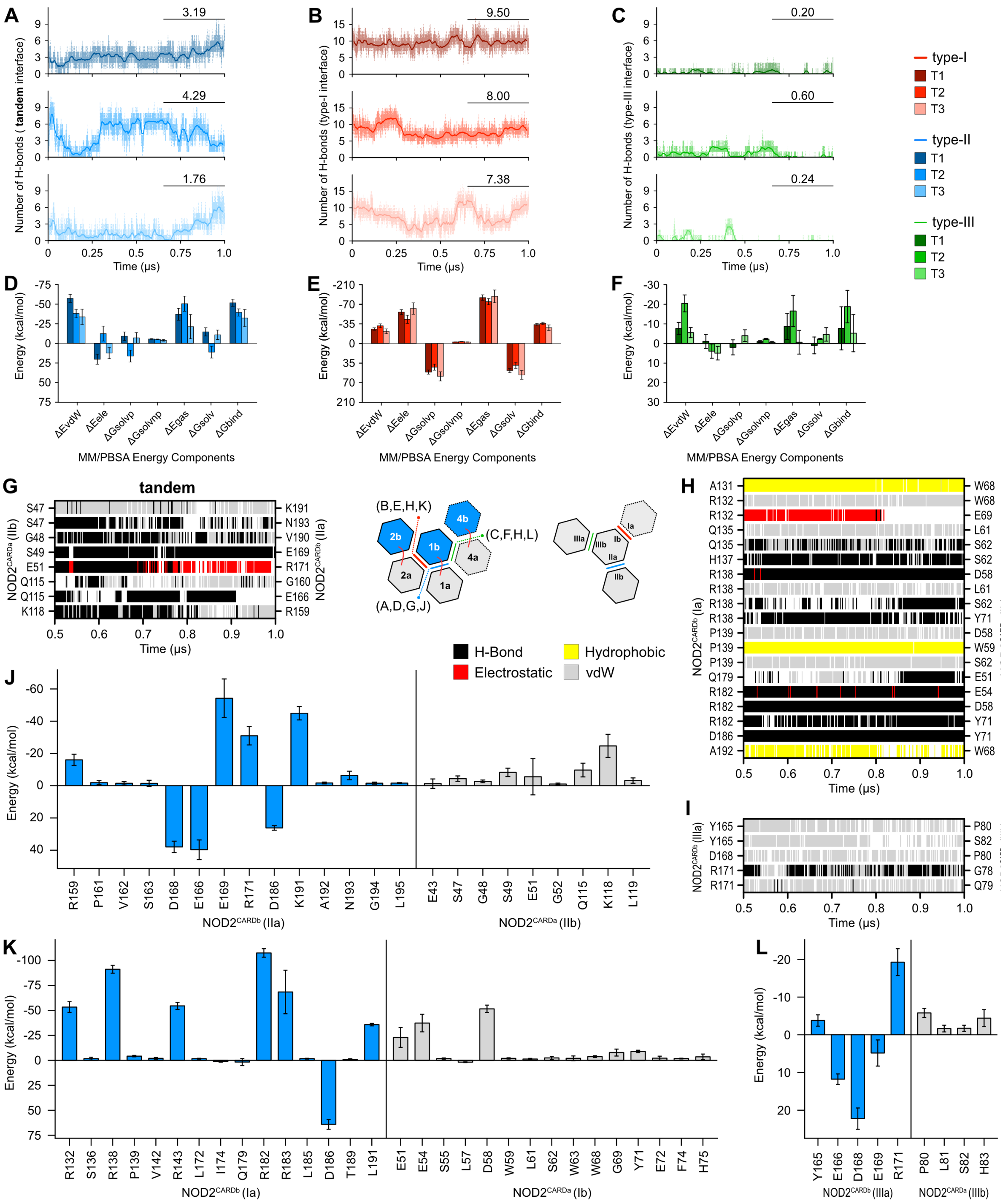

Fig. J. NOD2 CARDa–CARDb interactions within NOD2^{CARDab} rings. (A–C) Time evolution of H-bonds at the tandem (type-II) interface **(A)** and at the heterodimeric CARDa–CARDb type-I **(B)** and type-III **(C)** interfaces. **(D–F)** MM/PBSA binding free energies and their energy decompositions for the tandem type-II **(D)**, heterodimeric type-I **(E)**, and type-III **(F)** interfaces (Table G). **(G–I)** Residue–residue contact maps for representative tandem type-II **(G)**, type-I **(H)**, and type-III **(I)** heterodimers, showing H-bonds (black), electrostatic contacts (red), hydrophobic contacts (yellow), and van der Waals contacts (gray). **(J–L)** Per-residue MM/PBSA energy decomposition at the tandem type-II **(J)**, type-I **(K)**, and type-III **(L)** interfaces (Table H), highlighting energetic hotspots within CARDa and CARDb.

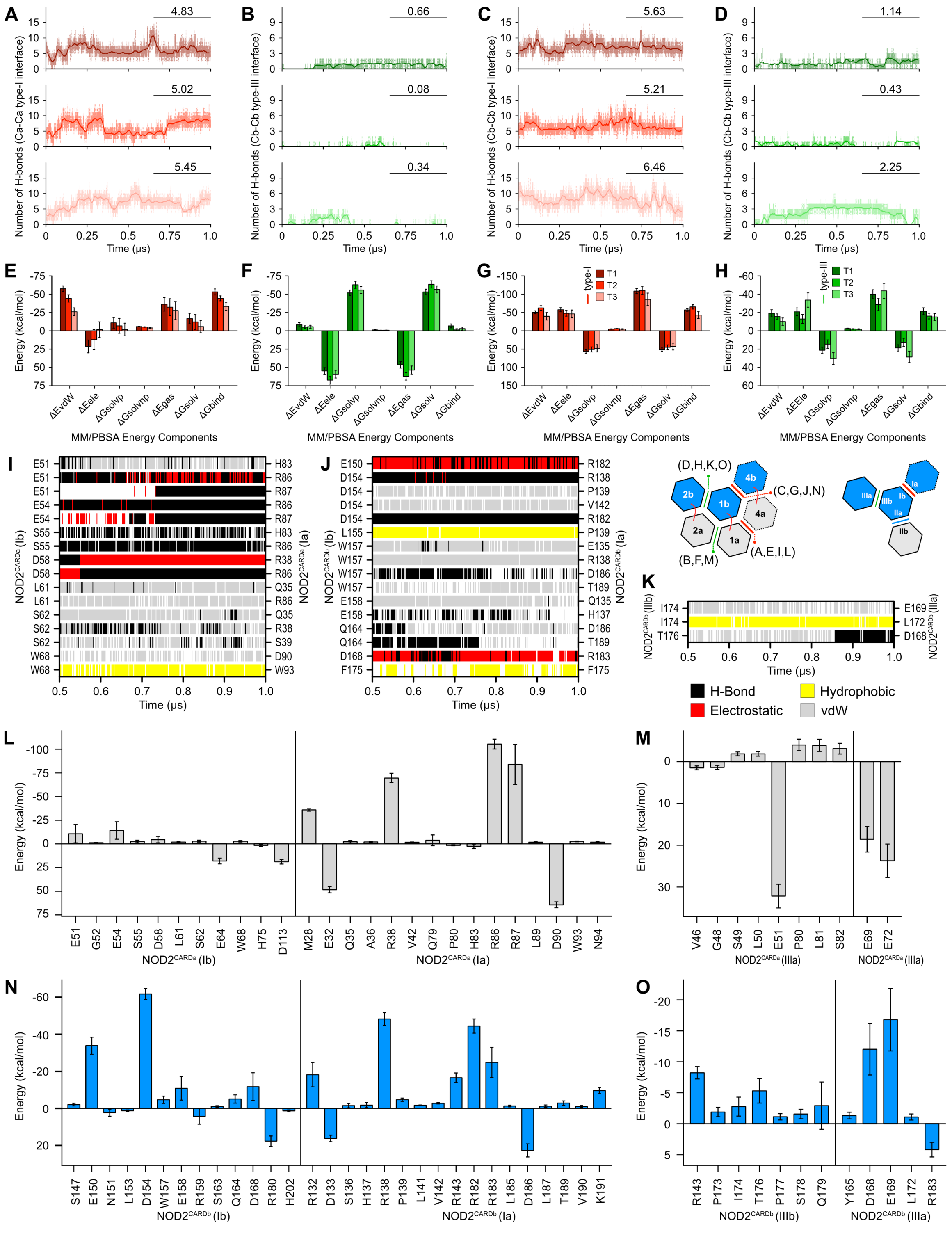

Fig K. Homodimeric interactions within tandem CARD rings in N2R2–F4 filament model. (A–D) Time evolution of intermolecular H-bonds at CARDa–CARDa **(A)** and CARDb–CARDb type-I **(B),** and type-III **(C)** interfaces. **(E–H)** MM/PBSA binding free energies and component contributions for CARDa–CARDa type-I **(E)** and type-III **(F)** and CARDb–CARDb type-I **(G)** and type-III **(H)** interfaces (see Table G). **(I–K)** Residue–residue contact maps for CARDa–CARDa type-I **(I)**, and CARDb–CARDb type-I **(J)** and type-III **(K)** interfaces. **(L–O)** Per-residue MM/PBSA energy decomposition for each homodimeric interface (Table H), identifying residues that support homotypic CARD contacts.

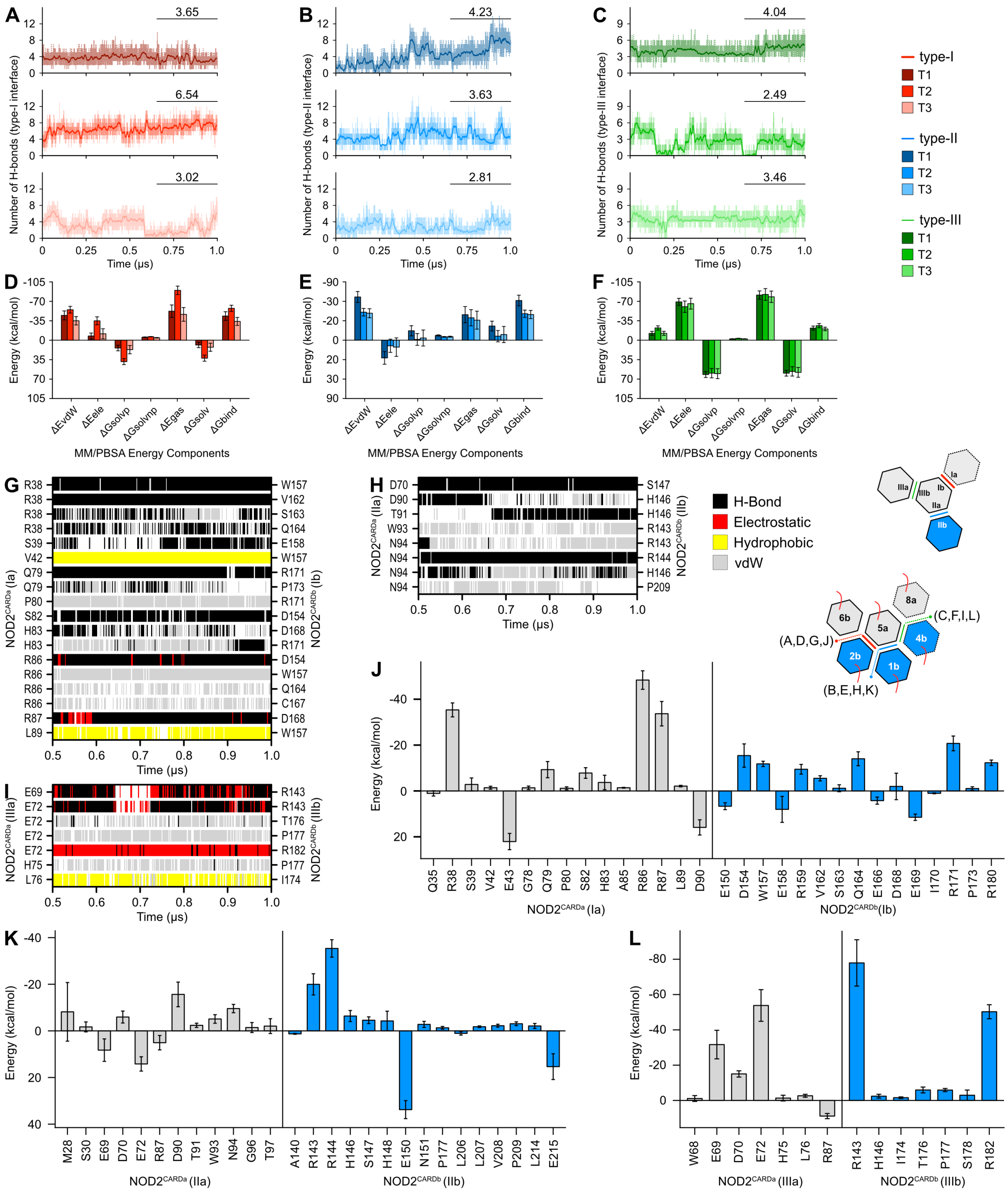

Fig L. CARDa–CARDb heterodimeric interfaces between adjacent NOD2^{CARDab} rings. (A–C) Time evolution of intermolecular H-bonds for type-I **(A)**, type-II **(B)**, and type-III **(C)** CARDa–CARDb interfaces between two NOD2^{CARDab} rings. **(D–F)** MM/PBSA binding free-energy components for type-I **(D)**, type-II **(E)**, and type-III **(F)** interfaces (Table G). **(G–I)** Residue–residue contact maps for representative type-I **(G)**, type-II **(H)**, and type-III **(I)** heterodimers, colored by contact type indicating H-bonds (black), electrostatic contacts (red), hydrophobic contacts (yellow), and van der Waals contacts (gray). (J–L) Per-residue MM/PBSA energy decomposition at type-I **(J)**, type-II **(K)**, and type-III **(L)** interfaces (see Table H), showcasing key residues that mediate inter-ring NOD2 CARDa–CARDb interaction.

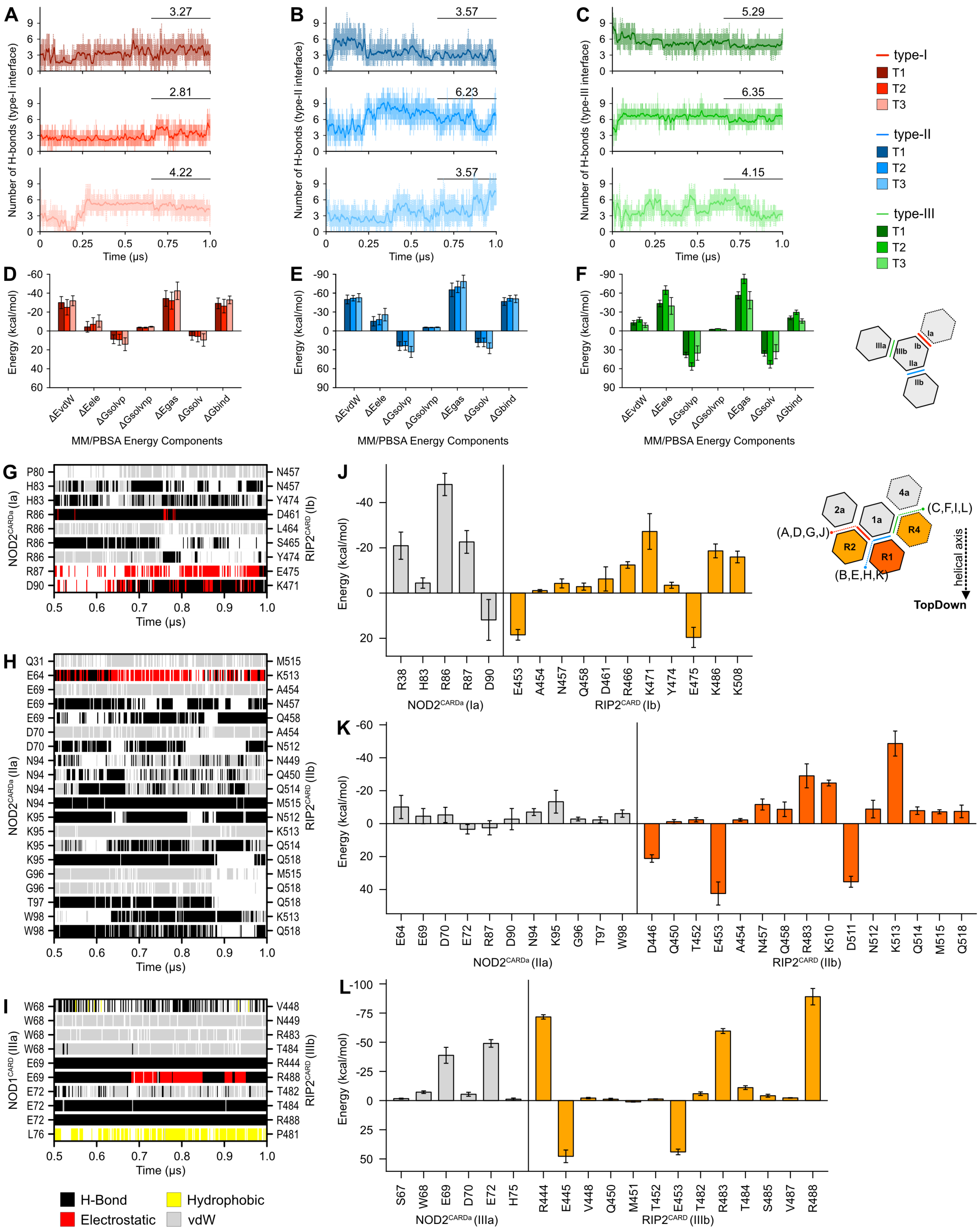

Fig M. Interaction dynamics at the NOD2^{CARDa}–RIP2^{CARD} top-down terminus. (A–C) Time evolution of intermolecular H-bonds for NOD2^{CARDa}–RIP2^{CARD} type-I **(A)**, type-II **(B)**, and type-III **(C)** interfaces over time. **(D–F)** MM/PBSA binding free-energy components for type-I **(D)**, type-II **(E)**, and type-III **(F)** interfaces (see Table G). **(G–I)** Residue–residue contact maps for representative type-I **(G)**, type-II **(H)**, and type-III **(I)** heterodimers. **(J–L)** Per-residue MM/PBSA energy decomposition for monomers participating in type-I **(J)**, type-II **(K)**, and type-III **(L)** interfaces (see Table H), highlighting energetic hot-spot residues at the top-down terminus.

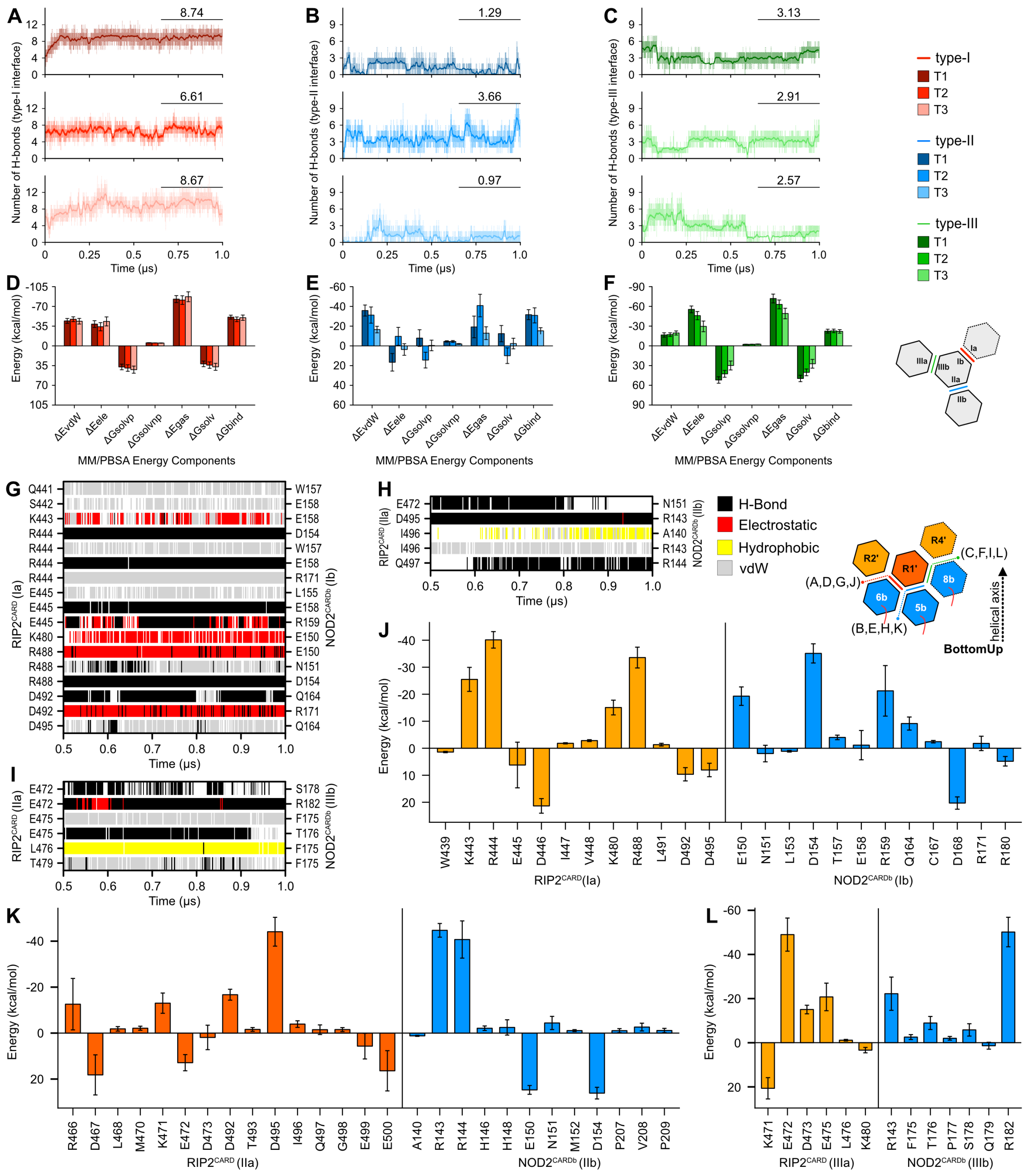

Fig N. Interaction stability and energetics at the NOD2^{CARDb}–RIP2^{CARD} bottom-up terminus. (A–C) Time evolution of intermolecular H-bonds for NOD2^{CARDb}–RIP2^{CARD} type-I **(A)**, type-II **(B)**, and type-III **(C)** interfaces across trajectories. **(D–F)** MM/PBSA binding free-energy components for type-I **(D)**, type-II **(E)**, and type-III **(F)** interfaces (see Table G). **(G–I)** Residue–residue contact maps for representative type-I **(G)**, type-II **(H)**, and type-III **(I)** heterodimers, with H-bonds (black), electrostatic contacts (red), hydrophobic contacts (yellow), and van der Waals contacts (gray). **(J–L)** Per-residue MM/PBSA energy decomposition for each CARD at type-I **(J)**, type-II **(K)**, and type-III **(L)** interfaces (see Table H), highlighting energetic hot-spot residues at the bottom-up terminus.

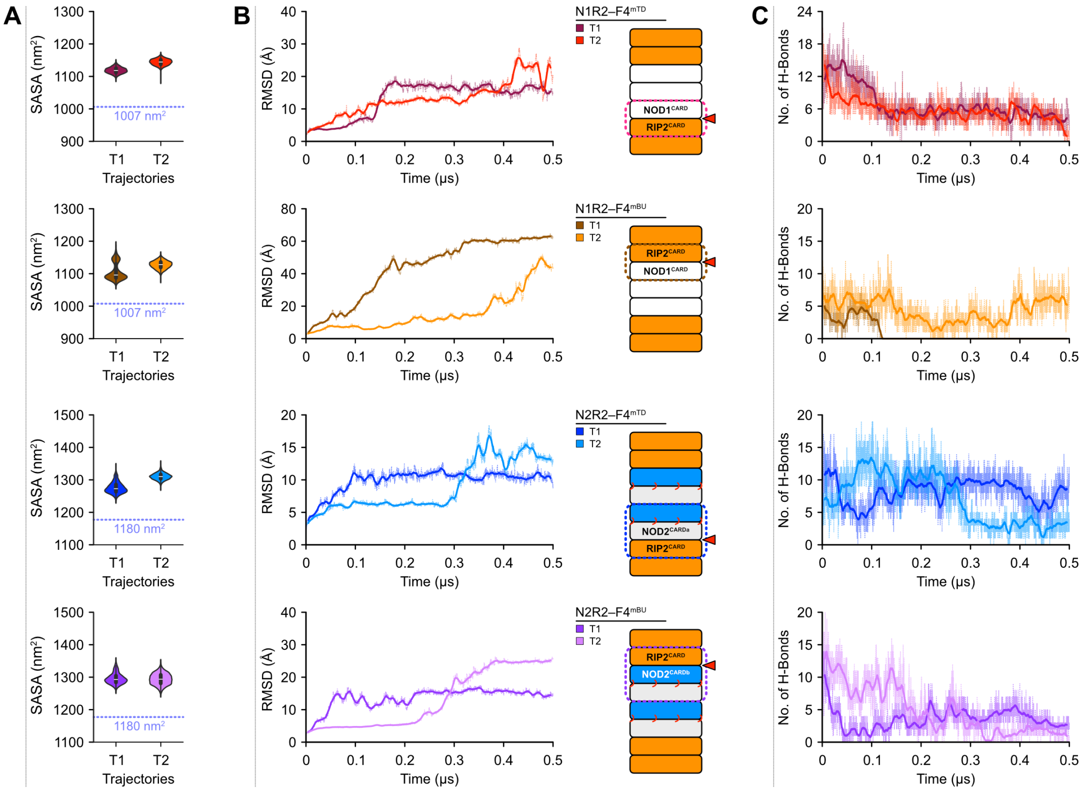

Fig O. Dynamic destabilization of mutant CARD filament constructs. (A) Violin plots of solvent-accessible surface area (SASA) for each mutant filament model (N1R2–F4^{mTD}, N1R2–F4^{mBU}, N2R2–F4^{mTD}, and N2R2–F4^{mBU}), showing SASA distributions across trajectories. Dashed lines indicate the SASA of the corresponding initial modeled complexes. **(B)** Backbone RMSD calculated for interfacial regions across two independent trajectories (T1 and T2). Insets indicate the location of the mutated interfaces within each filament model. **(C)** Total number of interfacial H-bonds over time at mutant interfaces.
